# Innate protection against intrarectal SIV acquisition by a live SHIV vaccine

**DOI:** 10.1172/jci.insight.175800

**Published:** 2024-05-21

**Authors:** Yongjun Sui, Thomas J. Meyer, Christine M. Fennessey, Brandon F. Keele, Kimia Dadkhah, Chi Ma, Celia C. LaBranche, Matthew W. Breed, Josh A. Kramer, Jianping Li, Savannah E. Howe, Guido Ferrari, LaTonya D. Williams, Maggie Cam, Michael C. Kelly, Xiaoying Shen, Georgia D. Tomaras, David Montefiori, Tim F. Greten, Christopher J. Miller, Jay A. Berzofsky

**Affiliations:** 1 Vaccine Branch and; 2 CCR Collaborative Bioinformatics Resource, National Cancer Institute, NIH, Bethesda, Maryland, USA.; 3 AIDS and Cancer Virus Program and; 4 Single Cell Analysis Facility, Frederick National Laboratory for Cancer Research, Frederick, Maryland, USA.; 5 Thoracic and GI Malignancies Branch, National Cancer Institute, NIH, Bethesda, Maryland, USA.; 6 Duke Human Vaccine Institute and; 7 Department of Surgery, Duke University School of Medicine, Durham, North Carolina, USA.; 8 Laboratory Animal Sciences Program, Frederick National Laboratory for Cancer Research, Bethesda, Maryland, USA.; 9 Duke Center for Human Systems Immunology, Duke University School of Medicine, Durham, North Carolina, USA.; 10 Center for Comparative Medicine, University of California, Davis, Davis, California, USA.

**Keywords:** AIDS/HIV, Vaccines, AIDS vaccine, Innate immunity

## Abstract

Identifying immune correlates of protection is a major challenge in AIDS vaccine development. Anti-Envelope antibodies have been considered critical for protection against SIV/HIV (SHIV) acquisition. Here, we evaluated the efficacy of an SHIV vaccine against SIVmac251 challenge, where the role of antibody was excluded, as there was no cross-reactivity between SIV and SHIV envelope antibodies. After 8 low-dose intrarectal challenges with SIVmac251, 12 SHIV-vaccinated animals demonstrated efficacy, compared with 6 naive controls, suggesting protection was achieved in the absence of anti-envelope antibodies. Interestingly, CD8^+^ T cells (and some NK cells) were not essential for preventing viral acquisition, as none of the CD8-depleted macaques were infected by SIVmac251 challenges. Initial investigation of protective innate immunity revealed that protected animals had elevated pathways related to platelet aggregation/activation and reduced pathways related to interferon and responses to virus. Moreover, higher expression of platelet factor 4 on circulating platelet-leukocyte aggregates was associated with reduced viral acquisition. Our data highlighted the importance of innate immunity, identified mechanisms, and may provide opportunities for novel HIV vaccines or therapeutic strategy development.

## Abstract 2

Live SHIV vaccine does not require adaptive immunity to confer protection, and such an approach could be applied to future HIV vaccine development.

## Introduction

Identifying immune correlates of protection is crucial for HIV vaccine development. In the RV144 trial, the only human HIV vaccine phase III clinical trial that showed significant protective efficacy (31%), the protection was associated with the IgG antibody responses against the V1V2 region of the HIV-1 envelope protein (1, 2). To test whether RV144-like non-neutralizing antibodies could mediate protection against viral acquisition, adoptive transfer experiments have been performed in macaque models. Disappointingly, only limited or no protection was found against SIV/HIV (SHIV) viral challenges in contrast with the strong protection by neutralizing antibodies (3–6). It is generally accepted that non-neutralizing antibodies, which lack antigen-dependent cellular cytotoxicity (ADCC) breadth, cannot mediate protection against viral acquisition. This motivates a search for immune mechanisms of protection other than anti-envelope (anti-Env) antibodies. In line with this, we found that a mucosal vaccine comprising modified vaccinia Ankara (MVA) and a recombinant envelope-CD4 fusion construct could reduce risk of SHIV viral acquisition (44% efficacy) in the absence of anti-Env antibody responses, implying the involvement of cellular and/or innate immunity (7).

As it is difficult to identify the protective cellular and/or innate immunity in a vaccine with only 30%–40% efficacy, we turned to live AIDS vaccines. Though live attenuated SIV vaccines will never be used as an HIV vaccine strategy due to safety concerns, they have been the most efficacious of the vaccine strategies tested in macaque models to date. Data from various labs showed that vaccination of rhesus macaques with SIVΔnef can induce robust immune responses to protect most of the vaccinated animals from intravenous or mucosal challenge with homologous or heterologous viruses (8–10). Among these findings, some with antibody-mismatched envelope still mediated protection against acquisition (8–10). Since the mismatch was only partial, these studies cannot exclude the possibility that antibodies against the matched part of the envelope played a role in mediating protection. To rule out this possibility, an SHIV live vaccine was shown to protect against intravaginal SIV challenge (11–15). It was found that about 60% of macaques infected with virulence-attenuated virus SHIV89.6 were protected from subsequent intravaginal pathogenic SIVmac239 challenge (11, 12). Due to the limitation of the technology at that time, the definition of protection was the ability of an animal to maintain plasma viral RNA levels below 10^4^ copies/mL plasma for 6 months postchallenge. Now with the deep sequencing technology, by which SHIV can be distinguished from SIV with as low as a few copies of viruses, we revisited the live SHIV vaccine study and evaluated the roles of CD8^+^ cell responses and/or innate immunity in mediating sterile protection against intrarectal SIV acquisition in the absence of anti-Env antibody responses. Our results showed that CD8^+^ cells were not essential for protecting against viral acquisition. To search for immune correlates of innate immunity, we found an immune tolerance signature with lower expression of genes in the interferon pathway and responses to virus/cytokine pathways in the protected animals. In addition, higher expression of platelet factor 4 (PF4) on the circulating platelet-leukocyte aggregates of the protected animals was correlated with reduced infection risk. Platelets represent a less well-recognized component of immunity. The data demonstrated the crucial roles of vaccine-induced innate immunity, including platelet-related activity, for protection against SIV viral acquisition.

## Results

### Significant protection is achieved against intrarectal SIV acquisition by a live SHIV vaccine in the absence of anti-Env antibody responses.

**Figure 1:**
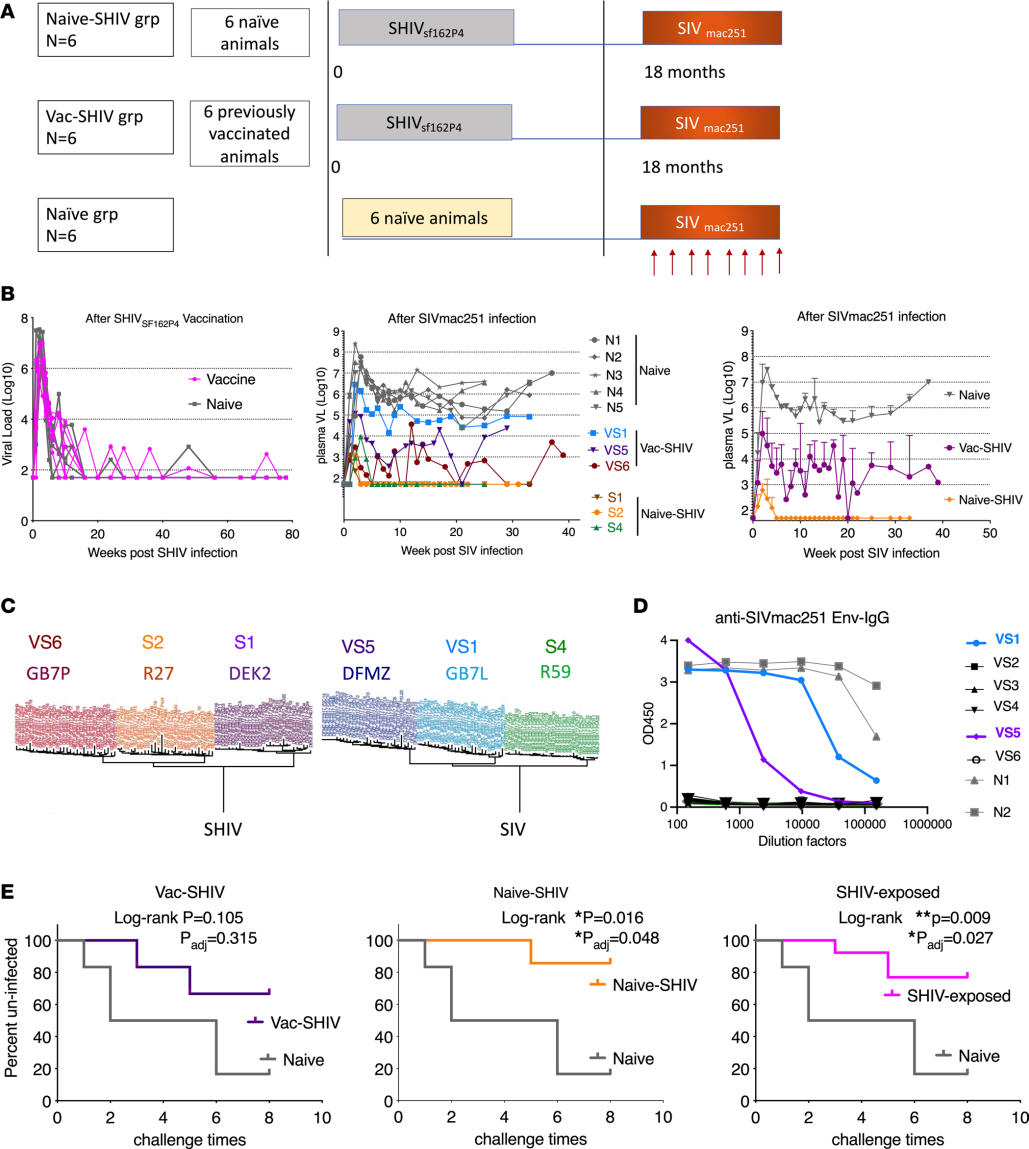
Study outline of SHIV vaccine and the viral load outcome after intrarectal, repeated, low-dose SIVmac251 challenges in rhesus macaques. (**A**) Schematic illustration of the vaccination and challenge protocol for the 3 groups of animals. (**B**) Viral loads (VLs) after SHIV vaccinations (*n* = 6 for vac-SHIV and *n* = 6 for naive-SHIV) and SIV infections (*n* = 11). The 7 animals (VS2, VS3, VS4, S3, S5, S6, and N6) that did not show detectable VLs are not shown in the middle and right panel. Mean ± SEM are shown. (**C**) Envelope sequencing tree of the animals with detectable VL after SIV challenges. (**D**) Anti-SIVmac251 Env IgG titers in serum samples collected 1 month after the last SIVmac251 challenges. Naive#1 and 2 were samples from macaques with confirmed infection with SIV. (**E**) SIV-uninfected (SIV-free) curves of the animals from different groups. SHIV-exposed is the combination of vac-SHIV and naive-SHIV groups. In all 3 panels in **E**, the same 6 naive animals were used for comparisons. Kaplan-Meier curve analysis was performed after a series of 8 intrarectal (IR) SIV challenges. For multiple comparisons, Bonferroni-Dunn methods were used to calculate the adjusted *P* values. Log-rank *P* values and adjusted *P* values are shown.

Twelve SHIV_SF162P4_-infected macaques were used as the SHIV-vaccinated group (Supplemental Table 1; supplemental material available online with this article; https://doi.org/10.1172/jci.insight.175800DS1). Among them, 6 were previously exposed to our mucosal HIV vaccine comprising full-length single chain fusion construct of HIV envelope with CD4, plus peptides and recombinant MVA in our previous study (7) (designated as the vac-SHIV group), and the rest of them were naive before SHIV challenge in the previous study (7) (designated as the naive-SHIV group, Figure 1A). In that earlier study, the vaccinated group required more challenges to infect with SHIV (Figure 3B of ref. 7), but once infected, both groups showed identical SHIV viral load (VL) and time course of gradual clearance (Figure 1B). Four months after SHIV infection, all 12 animals controlled their VLs and had no detectable VL within the limit of detection (50 copies/mL), except for occasional small transient blips (Figure 1B). As in the previous study, we observed that myeloid-derived suppressive cells (MDSCs) played an important role in affecting the challenge outcome. Here, we measured the MDSCs of these 12 animals 1 month before SIV challenge. Consistent with the absence of VLs in these animals, neither group had increased MDSC levels compared to naive controls, and no significant difference was found between the vac-SHIV and naive-SHIV groups (Supplemental Figure 1). Therefore, all 12 animals could be used in the current study, having had a similar single prior SHIV infection that now served as SHIV vaccination. Consistent with the fact that HIV and SIV Envelopes are completely distinct, no anti-SIV Env antibodies were detected in the SHIV-exposed animals before SIV challenge. This ruled out the possible contributions of anti-SIV Env antibody response. Then we challenged these macaques along with 6 naive macaques with low-dose, weekly, intrarectal (IR) SIVmac251 for 8 weeks (Figure 1A). After 8 challenges, 5 out of 6 naive controls were infected with SIVmac251 infection, with log peak VL 5.7 copies/mL and set point VL 4.2 copies/mL (Figure 1B). No significant difference was observed between Mamu A*01^+^ and A*01^–^ macaques in the VLs before and after SIVmac251 infection. Three animals in vac-SHIV and 3 animals in naive-SHIV groups remained uninfected (Figure 1B). For the remaining 6 animals in vac-SHIV and naive-SHIV groups, to assess whether the VLs were from SIV infection or from SHIV rebound, we performed single genome sequencing for envelope genes. Three of them (GB7P/VS6, R27/S2, and DEK2/S1) turned out to have rebounded with SHIV, while the remaining 3 (DFMZ/VS5, GB7L/VS1, and R59/S4) were infected by SIV (Figure 1C). We also measured the anti-SIVmac251 Env IgG responses in the serum collected 1 month after the last viral challenge. Among the 3 SIV-infected macaques, GB7L/VS1 and DFMZ/VS5 had SIVmac251 seroconversion, while R59/S4 did not (Figure 1D). To summarize the challenge outcome (Figure 1E), 4 out of 6 animals in the vac-SHIV group (*P* = 0.1) and 5 out of 6 in the naive-SHIV group (*P* = 0.02) were protected. The protection against SIVmac251 acquisition did not differ significantly between the Mamu A*01^+^ and A*01^–^ macaques either. Overall, the SHIV-vaccinated group (*n* = 12, with 9 protected) were significantly different from the naive group (with 1/6 uninfected), with 83% vaccine efficacy (*P* = 0.009).

**Figure 2:**
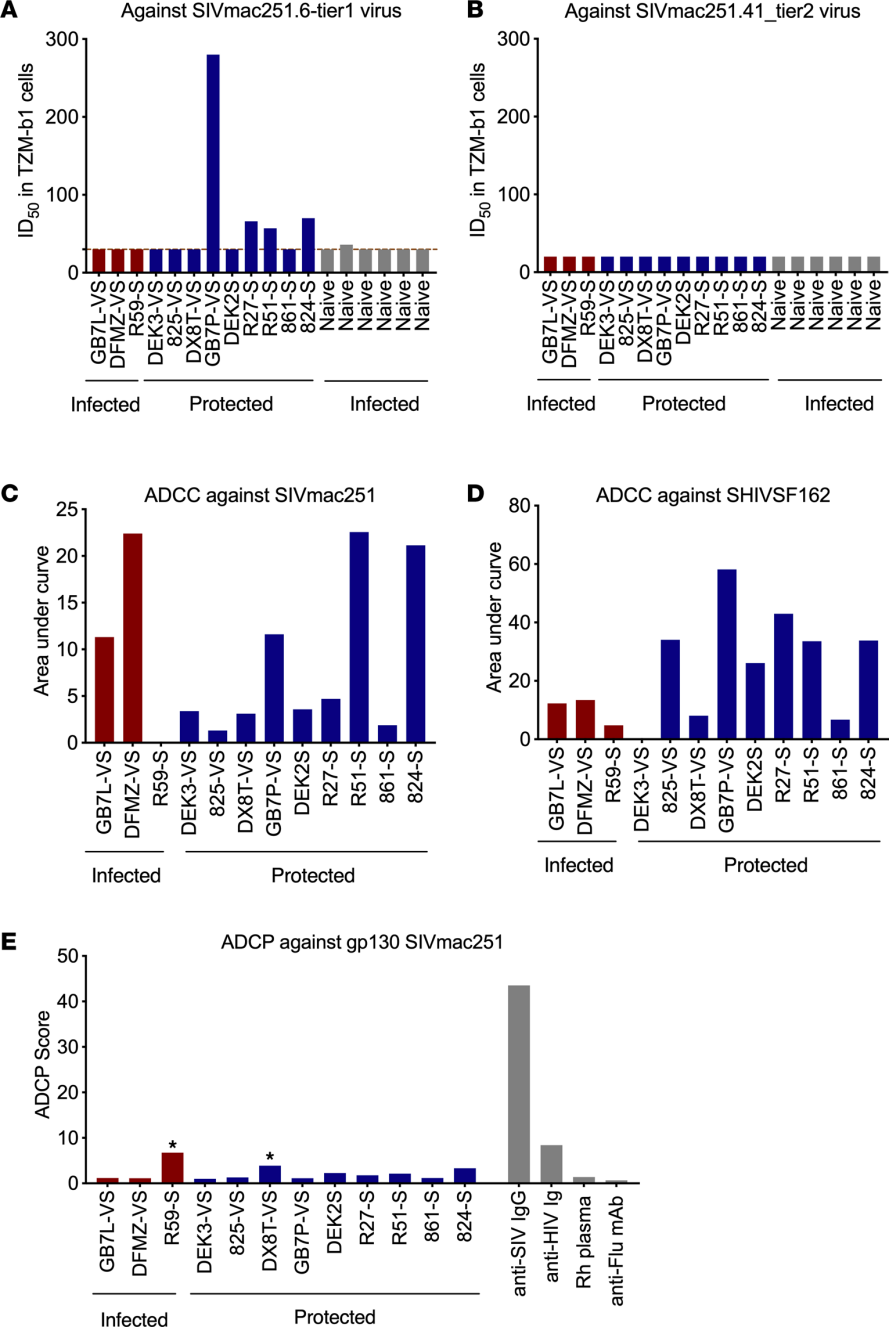
Viral neutralization and ADCC and antibody-dependent cellular phagocytosis titers in serum samples collected before SIVmac251 challenge. The neutralization titers against tier 1 and 2 SIVmac251 virus (**A** and **B**), ADCC titers against SIVmac251 and SHIVSF162 (**C** and **D**), and antibody-dependent cellular phagocytosis (ADCP) against gp130 SIVmac251 (**E**) were measured. For neutralization assays, values are the serum dilution at which relative luminescence units (RLUs) were reduced 50% compared with virus control wells (no test sample), while for ADCC and ADCP assays, values are the AUC and ADCP scores. *Indicates positive responders in the ADCP assay (**E**).

Since no anti-SIV Env antibodies were detected before challenge, we next evaluated other serum protective factors, such as antibodies against other viral proteins and/or chemokines in the plasma/serum, that might mediate protection. Using neutralization assays, we detected neutralizing antibody activity against HIV SF162 in all samples except DEK3, for which the activity was similar to that in the preimmunizatopm sample. Four animals showed weak serum neutralizing activity in the postimmunization samples against the tier 1 clone SIVmac251.6 virus, GB7P/VS6, R27/S2, R51/S3, and 824/KMV/S6 (Figure 2A). We did not detect any neutralizing activity against the tier 2 clone SIVmac251.41 (Figure 2B). These data suggested that protective factors in the serum cannot explain the SIV challenge outcomes (as the challenge virus SIVmac251 is a swarm containing both tier 1 and tier 2 viruses). We further assessed the antibody functionality by ADCC and antibody-dependent cellular phagocytosis (ADCP) assays. There were no significant differences in the responses of infected and protected animals regardless of whether anti-SIVmac251 or SHIVSF162 was analyzed (Figure 2, C–E). This finding along with the challenge outcome data verified the substantial protection in the absence of anti-Env antibody responses.

### Viral-specific T cell responses are induced but do not correlate with protection.

**Figure 3:**
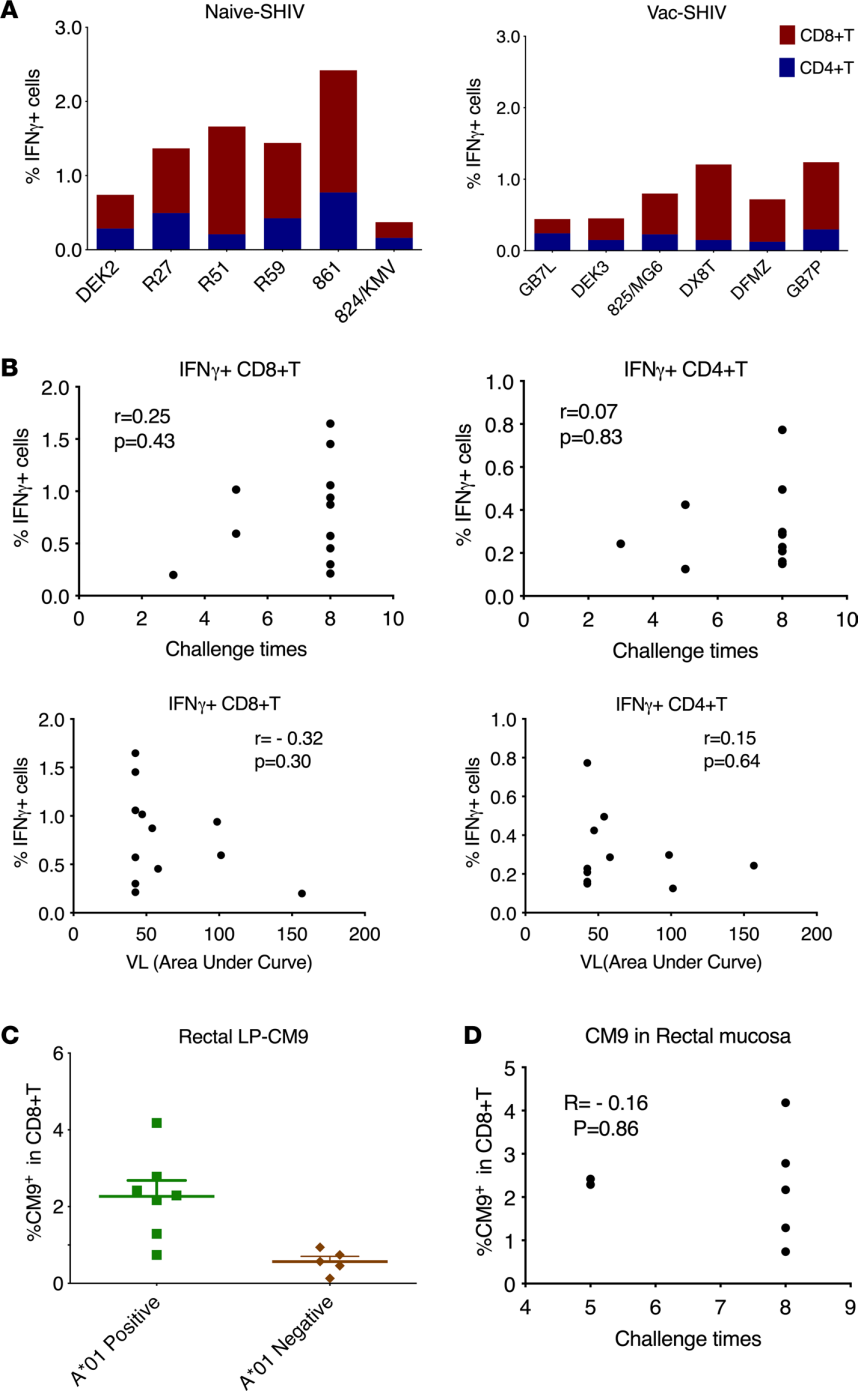
Viral-specific T cell responses in blood and rectal mucosa 1 or 2 months before the SIVmac251 viral challenges. (**A**) Intracellular staining of interferon-γ (IFN-γ) on CD8^+^ T and CD4^+^ T cells of the PBMCs was measured after overnight stimulation with overlapping peptide pools of Gag and Tat. (**B**) The correlation between the viral-specific CD8^+^ T/CD4^+^ T cells in the PBMCs and the SIV viral challenge numbers/viral load (AUC). (**C**) Gag-dominant CM9-dextramer^+^ responses in the rectal lamina propria (LP) were assessed in the SHIV-vaccinated animals. (**D**) The correlation between the CM9-dextramer^+^ responses in the rectal LP and the SIV viral challenge numbers. Both PBMC and rectal mucosa samples were collected 1–2 months before SIV challenges. Spearman’s correlation *R* and *P* values are shown.

Viral-specific T cell responses were evaluated by measuring the intracellular IFN-γ responses. As the envelopes from SHIV and SIV have no cross-reactivity, we included only Gag and Tat peptides to assess the viral-specific T cell responses. Low- to mid-level viral-specific CD4^+^ and CD8^+^ T cell responses were induced in the PBMCs of the SHIV-vaccinated animals (Figure 3A). However, none of them correlated with number of viral exposures required for the animals to be infected or VL (Figure 3B). The viral-specific T cell responses were 0.3% ± 0.07% for CD4^+^ T and 0.8% ± 0.2% for CD8^+^ T in the protected animals, comparable to those in the unprotected animals (0.3% ± 0.09% and 0.6% ± 0.2% for CD4^+^ and CD8^+^ T cells, respectively). Gag-specific mucosal T cell responses in the rectal mucosa were measured using CM9-dextramer. Among the Mamu*A01^+^ animals, the SHIV-vaccinated animals had 2.3% ± 0.4% of CM9-dextramer^+^ cells within CD8^+^ T cells in the rectal mucosa (Figure 3C). However, these responses did not correlate with number of viral exposures required to infect either (Figure 3D). Thus, viral-specific T cell responses were induced in the blood and rectal mucosa of the SHIV-vaccinated animals. However, they did not correlate with protection, suggesting that viral-specific T cell responses may not play an important role in reducing viral acquisition.

### Administration of anti-CD8α antibody achieves complete and prolonged depletion of systemic and mucosal CD8^+^ cells.

To better assess whether CD8^+^ cells play a role in mediating protection against SIV challenge, we conducted 2 CD8 depletion studies using MT807R1 antibody targeting CD8α chain, which has been shown to be able to deplete CD8^+^ cells in the blood completely (16–20). The peripheral CD8 depletion has been well characterized (16–20). However, the mucosal CD8 depletion kinetics are less described. To evaluate the CD8^+^ cell depletion in gut mucosal tissues, we first did a pilot study to characterize the CD8^+^ cell kinetics in rectal mucosa and blood (Supplemental Figure 2). Consistent with previous reports (16–20), complete CD8 depletion in the blood started from as early as day 4 and was persistent until day 14. On day 17, the CD8^+^ cells recovered in 2 out of the 6 animals (Supplemental Figure 2B). CD4^+^ T cell number was increased in blood 28 days posttreatment (Wilcoxon *P* = 0.03 compared with day 0; Supplemental Figure 2). Coinciding with CD8 depletion in blood, plasma VLs rebounded from day 7 and lasted for about 2 weeks. With the recovery of CD8^+^ cells in blood, VLs were controlled, verifying that the VL control in these animals was due to CD8^+^ cells (Supplemental Figure 2C). In the rectal mucosa, the absolute number and the percentage of CD8^+^ cells were significantly reduced from week 1 to week 6 compared with pretreated levels, while there were no significant CD4^+^ cell number changes (Supplemental Figure 2D). Notably, a complete CD8^+^ cell depletion from week 1 to week 3 was observed in the rectal mucosa of several animals, and the levels were minimal in the others (Supplemental Figure 2D). This 2-week window of time allowed us to evaluate the roles of CD8^+^ cells in preventing SIV acquisition.

### CD8^+^ cells are not necessary for protection against SIV acquisition in SHIV-vaccinated animals.

**Figure 4:**
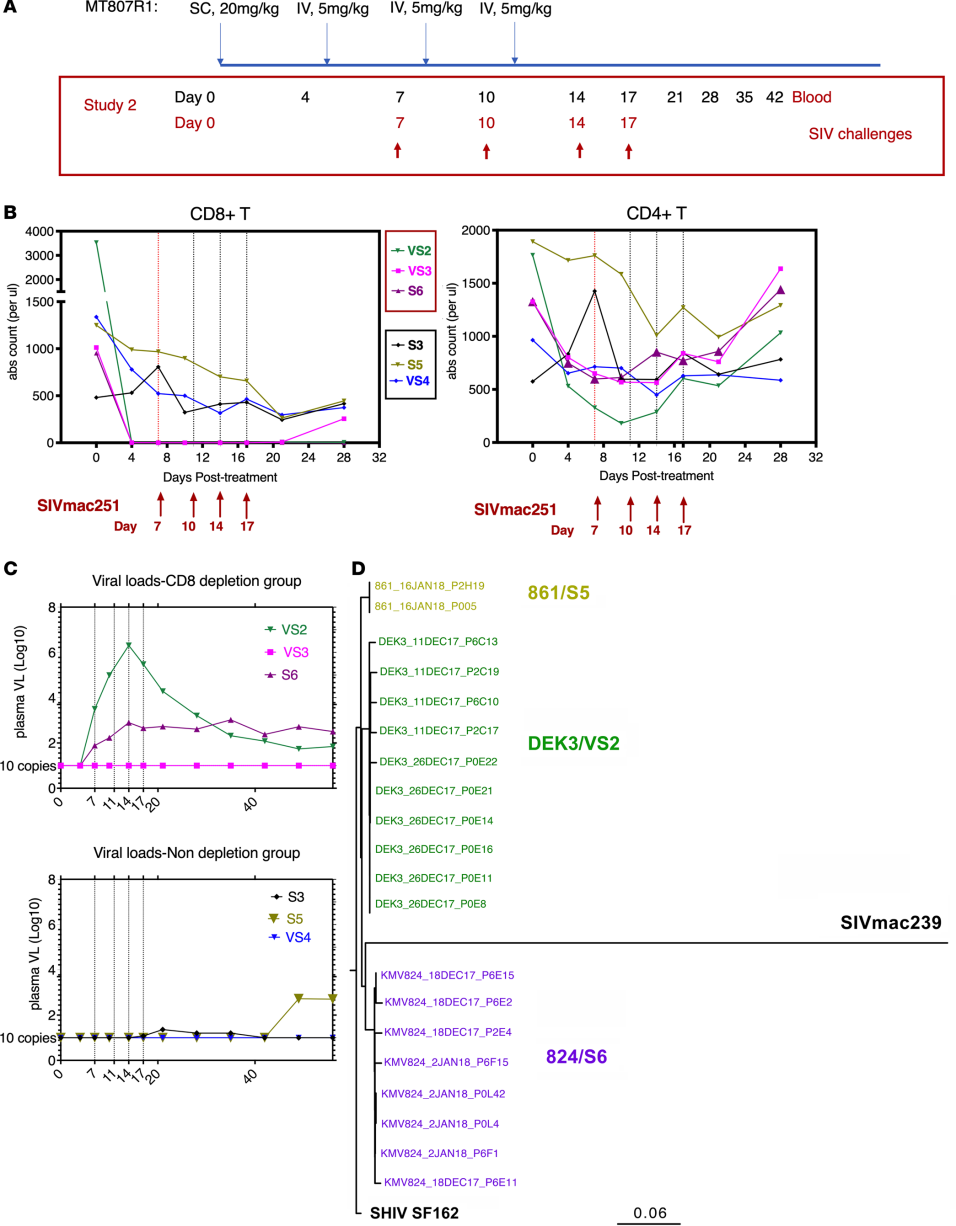
The CD8^+^ and CD4^+^ cells in PBMCs, as well as plasma viral loads, after administration of an anti-CD8α antibody MT807R1 followed by 4 times of SIVmac251 viral challenges. (**A**) Schematic diagrams of CD8 depletion using MT807R1 antibodies. (**B** and **C**) The dynamics of CD8^+^ and CD4^+^ T cells (**B**), and viral loads (**C**), in the peripheral blood after administration of CD8 antibody followed by 4 times of SIV challenge. Three animals — VS2, VS3, and S6 — received anti-CD8 depletion, while the other 3 animals — S3, S5, and VS4 — received PBS. (**D**) Envelope sequencing of the SHIV/SIV in the 3 animals showing viral loads. The cutoff threshold for viral load measurement is 10 copies/mL.

To make the mucosal CD8 depletion more efficient, we included an IR administration at day 14 in addition to the suggested standard CD8 depletion approach (1 subcutaneous + 3 intravenous [IV] administrations) for the pilot study. We found that even without IR administration, the mucosal CD8 depletions at week 1 and week 2 were very efficient. For the CD8 depletion SIVmac251 challenge study, 6 SHIV-vaccinated animals that had never shown VLs in the plasma after SIV challenge were included (Figure 4A). Three of them (S6, VS3, and VS2) underwent CD8 depletion, while the rest of them (S5, VS4, and S3) were controls receiving only PBS. In the first pilot study, the blood CD8^+^ T cells started to recover at day 17, suggesting that IR administration was not effective to deplete blood CD8^+^ T cells. Thus, for the CD8 depletion SIVmac251 challenge study, we included an extra IV (5 mg/kg) instead of IR administration at day 14. Indeed, we found that the CD8^+^ cells in the blood were completely depleted from day 4 to day 21 in the CD8 depletion group, while CD4^+^ T cells were not severely affected during this period (Figure 4B).

Four identical doses of SIVmac251 IR challenge, administered on days 4, 10, 14, and 17 after starting the anti-CD8 antibody treatment, were given to all 6 animals as before (Figure 4A). Two animals in the depletion group and 1 animal in the nondepletion group showed Gag VL (Figure 4C). Sequencing data demonstrated that the virus spikes were all rebound SHIVs, and none of these animals was infected by SIVmac251 (Figure 4D). Thus, the depleted group remained protected from SIVmac251 despite the absence of CD8^+^ cells. These data showed that CD8^+^ cells were not necessary for the reduction of viral acquisition, consistent with the recent study using a live attenuated SIV vaccine (21).

### Innate immunity factors associated with protection.

To identify mechanisms of protective innate immunity, we did an RNA-Seq analysis using isolated RNAs from unstimulated PBMC samples (designated as O samples) as well as PBMC and myeloid cell samples stimulated with SIVmac251 for 18 hours (designated as P and M samples, respectively) (Supplemental Figure 3A). The latter 2 samples were used to assess the immediate global responses upon viral exposure. The rationale for studying myeloid cells and PBMCs after ex vivo SIV stimulation was that adaptive immunity did not seem to play an important role here, and we (7, 22) and others (23, 24) have previously found that myeloid cells contribute to innate protection against SIV/SHIV acquisition. PBMCs rather than purified T cells were included as the interaction between myeloid cells and other cell types might also affect the challenge outcome. All the samples were collected before the animals were challenged with SIVmac251, and the animals were free of any SHIV/SIV VLs. We included in the RNA-Seq analysis 3 naive (randomly selected N1, N2, and N3), 3 SHIV-vaccinated and later infected (VS1, VS5, and S4), and 3 SHIV-vaccinated and later protected animals (VS2, S5, and S6, randomly selected from the 6 animals that did not show either SHIV rebound or SIV infection) (Supplemental Figure 3A and Supplemental Table 1). Differentially expressed genes (DEGs) between protected and infected animals were identified in the 3 types of samples. The top overlapping DEGs included TRBV29-1, ESPL1, F5, GNAZ, TRBV19, and CACNA1H (Supplemental Figure 3, B and C). Two TCRβ genes, TRBV29-1 and TRBV-19, had higher expression levels in the protected animals compared with the infected ones. It has been shown that CD8^+^ T cells against the immunodominant epitope of influenza A virus preferentially used the public TRBV19/TRAV27 TCRαβ clonotypes and displayed highly polyfunctional and proliferative capacity (25–27). Whether TRBV29-1 and TRBV-19 are the preferential T cell Vβ clonotypes in the protected animals needs further investigation.

**Figure 5:**
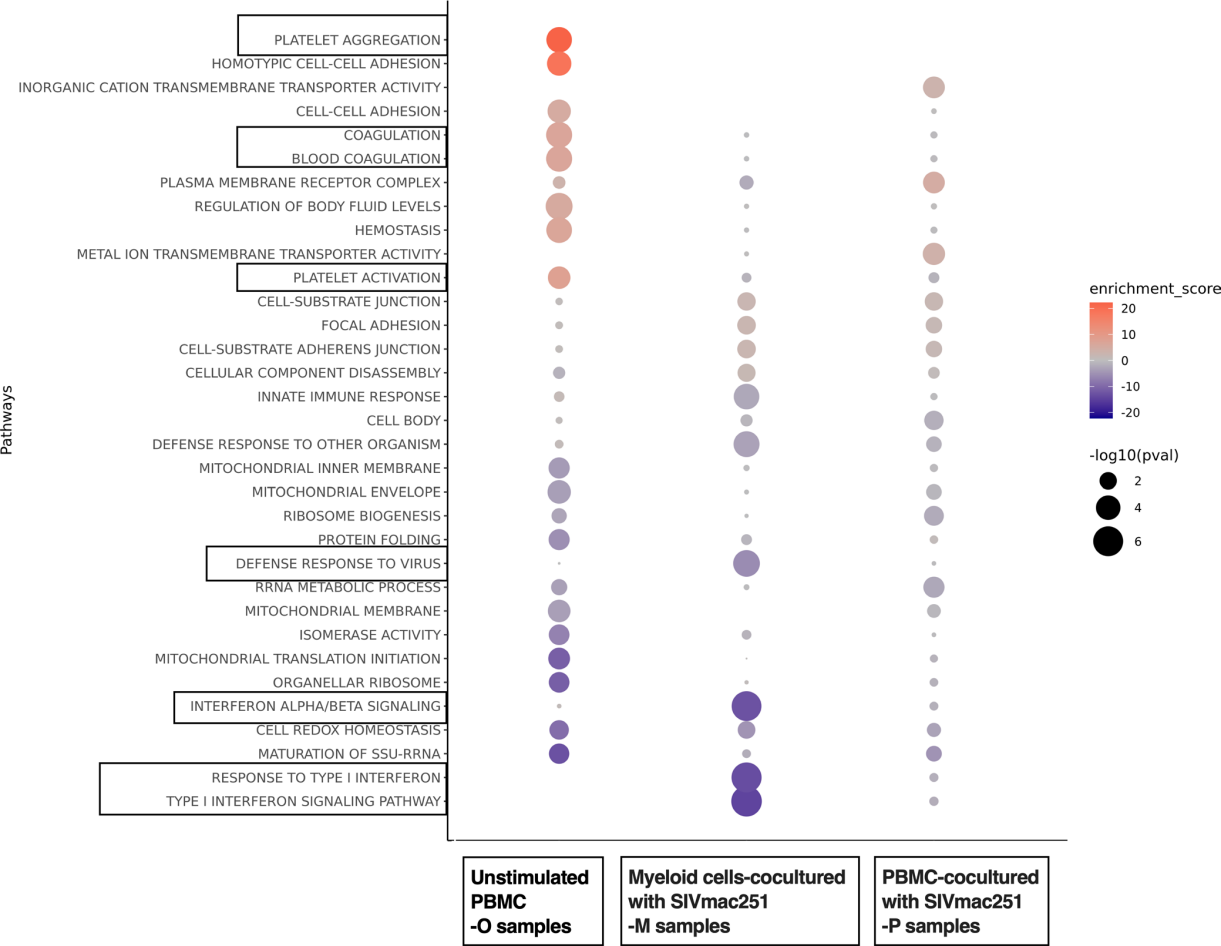
Pathway analysis of the differentially expressed genes between the protected versus the infected animals in 3 different sample types (samples were taken before SIVmac251 challenges). O samples: original PBMCs without any stimulation; P samples: PBMCs incubated/stimulated with SIVmac251 virus for 18 hours; M samples: enriched myeloid cells incubated/stimulated with SIVmac251 virus for 18 hours. The protected animals, from the SHIV-vaccinated group, were protected against later SIVmac251 infection, while the infected animals, 3 from the SHIV-vaccinated group and 3 from the naive group, were infected later after SIVmac251 challenges.

Gene ontology (GO) analysis, Kyoto Encyclopedia of Genes and Genomes analysis, Reactome analysis, and gene set enrichment analysis (GSEA) were performed to identify the upregulated and downregulated pathways involved (Figure 5 and Supplemental Table 2). In the protected animals, interferon-α, -β, and -γ signaling; responses to LPS, cytokines, and virus; and defense responses were among the downregulated pathways, while pathways including platelet aggregation/activation, coagulation, and cell-cell adhesion were upregulated (Figure 5 and Supplemental Table 2).

### Protected animals show an immune tolerance signature.

**Figure 6:**
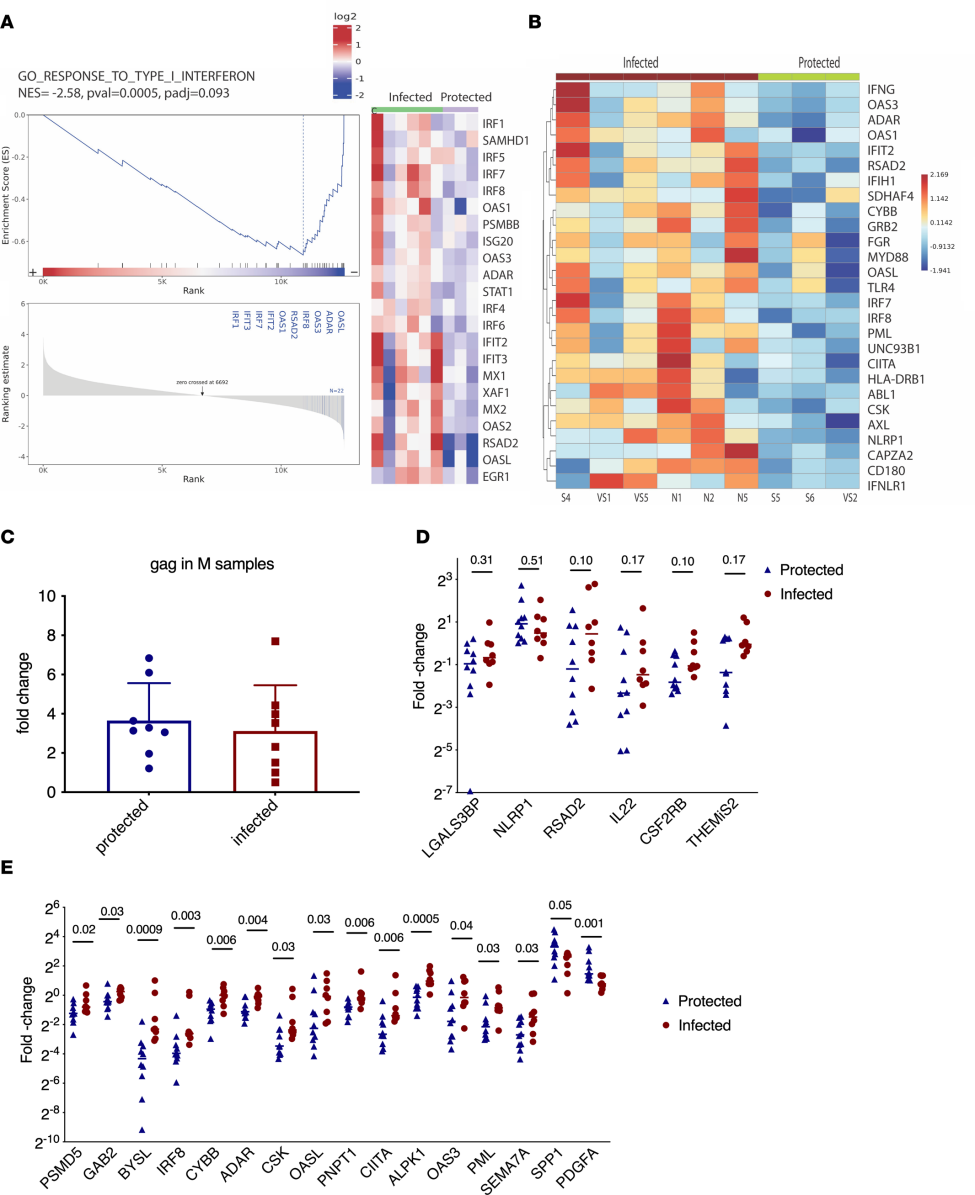
Vac-protected animals demonstrated an immune tolerance gene signature in the SIV-exposed myeloid cells. (**A**) Downregulated GSEA pathway (GO_ responses to type I interferon). The negative normalized enrichment scores indicate the downregulation of the pathways. (**B**) Heatmap of the downregulated gene pathway (innate immune responses GO 0045087). (**C**) The M (myeloid cell enriched) samples were incubated with SIVmac251 for 18 hours. RNA was isolated. Gag RNA level in the M samples from all 18 animals. (**D** and **E**) Quantitative PCR (qPCR) was used for validation of the down- and upregulated DEGs between the protected and infected animals (*n* = 18). Genes that were not significantly changed are shown in **D**, and those that were significantly changed are shown in **E**. These genes were involved in the following pathways: interferon alpha/beta/gamma signaling; response to LPS, cytokines; responses to virus; and influenza A. Two upregulated genes, SPP1 and PDGFA, were included as technical controls. Mann-Whitney was used for comparisons between the protected animals and the infected animals.

The data suggested reduced IFN-related or viral response–related pathways might be associated with protection (Figure 6, A and B). We isolated RNAs from myeloid cell–enriched cultures after 18 hours of ex vivo exposure to SIVmac251. The Gag expression levels were similar in the SIV-exposed samples, excluding the differential influence of VLs (Figure 6C). After measuring the mRNA expression levels of these pathway-related genes in all 18 animals, we validated that 14 out of 20 genes showed significantly lower expression levels in the protected animals compared with those of the infected ones (Figure 6, D and E). Furthermore, 15 out of these 20 genes, BYSL, ADAR, ALPK1, CIITA, CYBB, IRF8, NPT1, PSMD5, CSF2RB, CSK, GAB2, OAS3, OASL, PML, and SEMA7, were inversely correlated with the numbers of viral challenges (Supplemental Table 3). To figure out whether the downregulation signature of these pathways (Figure 6E) resulted from SHIV vaccination, we compared the gene expression levels of the myeloid cell–enriched cultures from SHIV-vaccinated animals to those of the naive animals, which were also collected about 1 month before SIV challenge. We found that the expression levels of these genes were lower in the SHIV-vaccinated animals compared with those of the naive animals irrelative of the later SIV challenge outcome, suggesting that the expression levels of these genes were decreased by SHIV vaccination (Supplemental Figure 4). We further assessed whether MHC haplotype (Mamu A*01) plays a role and did not find any significant difference between the expression levels of these genes in the Mamu A*01-positive and -negative animals, irrespective of vaccination (Supplemental Figure 5).

### Increased platelet-leukocyte aggregation with PF4 expression is associated with protection.

**Figure 7:**
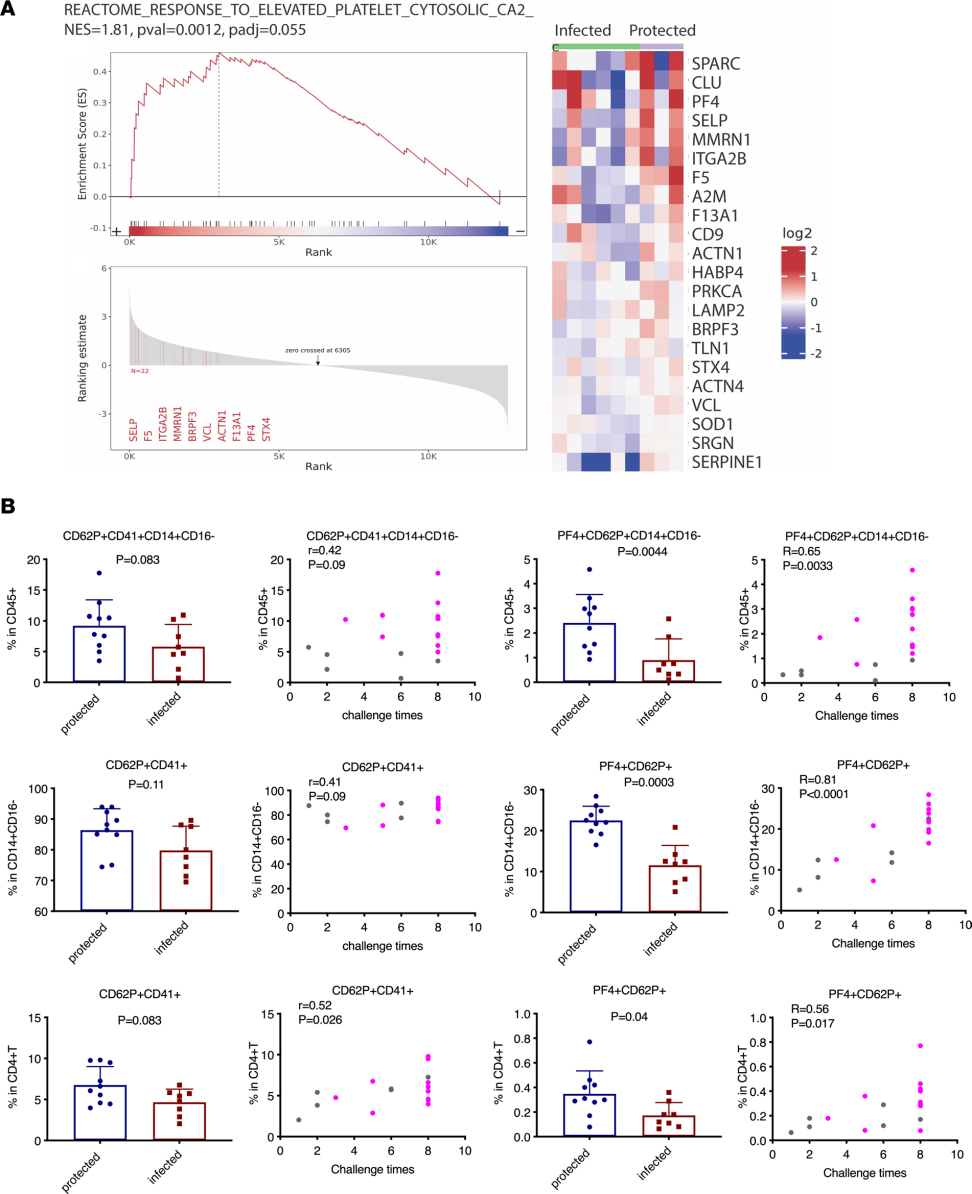
The platelet-related GSEA pathway and the frequencies of activated platelets and PF4 on monocytes and CD4^+^ T cells. (**A**) Heatmap and GSEA pathway (Reactome response to elevated platelet cytosolic) in the PBMCs of the protected and infected animals. The positive normalized enrichment scores indicate the upregulation of the pathways. (**B**) Flow cytometry analysis of activated platelet and PF4 expression on monocytes and CD4^+^ T cells in the PBMCs of all 18 animals. Mann-Whitney analysis was used for comparisons between the protected and infected animals. The Spearman’s correlations between the frequencies of platelet-leukocyte aggregate and challenge times were analyzed; *R* and *P* values are shown. The PBMC samples were collected 1 month before SIVmac251 challenges. In the correlation panels, gray dots show the naive animals, while magenta dots show the vaccinated animals.

Pathways related to platelet aggregation/activation, with altered genes, such as P2RY12, F5, TUBB1, VCL, GP1BA, ITGA2B, GP9, GP1BA, P2RX1, and PTGS1, were involved (Figure 7A). To figure out whether platelet-leukocyte aggregates and PF4 were associated with viral protection, we measured their frequencies in the PBMCs 1 month before SIVmac251 viral challenge (gating strategies in Supplemental Figure 6). Monocyte-platelet aggregates (MPAs) were detected: the CD14^+^CD16^–^ subset had the highest frequencies of CD41^+^CD62P^+^ indicators of bound activated platelets followed by the CD14^+^CD16^+^ and CD14^–^CD16^+^ subsets. While the difference between the protected and the infected animals was not so prominent (was a trend) for the frequencies of CD62P^+^CD41^+^ on CD14^+^CD16^–^ monocytes and CD4^+^ T cells, the frequencies of PF4^+^CD62P^+^ on these 2 cell subsets were strikingly different (Figure 7B). These should best mediate protection. The protected animals had higher frequencies of CD62P^+^CD41^+^ on CD14^+^CD16^–^ monocytes and CD4^+^ T cells, which were positively correlated with the number of viral challenges required to acquire SIV (Figure 7B). The strongest correlations were with aggregates containing PF4 (Figure 7B). The data suggested that the PF4 on the surface of CD14^+^CD16^–^ MPAs and CD4^+^ T cells might contribute to antiviral protection against SIVmac251 challenge. Interestingly, we did not find any effect of MHC haplotype (Mamu A*01) on platelet aggregates on monocytes or CD4^+^ T cells (Supplemental Figure 7).

### The biomarkers of MPAs in PBMCs.

**Figure 8:**
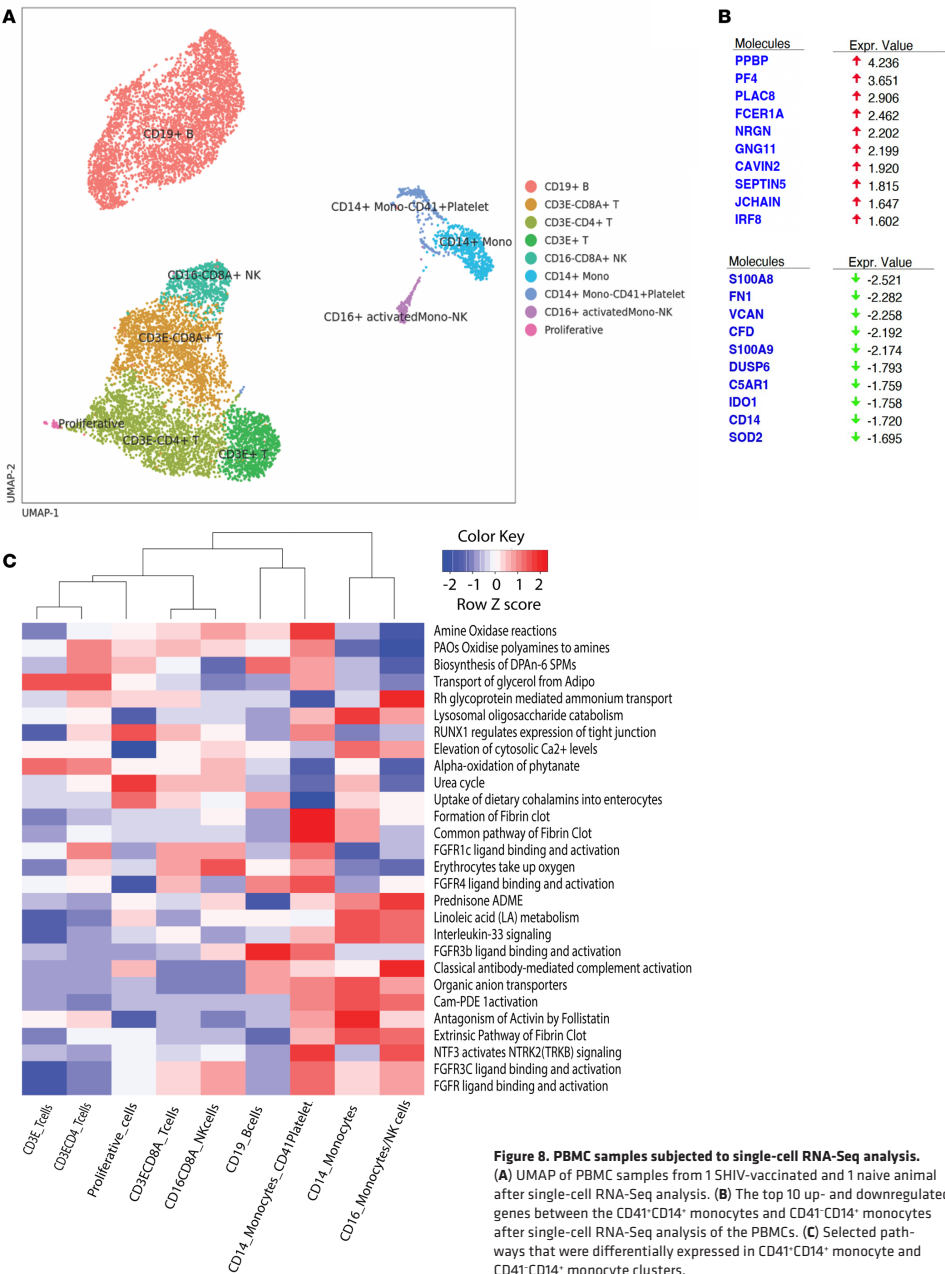
PBMC samples subjected to single-cell RNA-Seq analysis. (**A**) UMAP of PBMC samples from 1 SHIV-vaccinated and 1 naive animal after single-cell RNA-Seq analysis. (**B**) The top 10 up- and downregulated genes between the CD41^+^CD14^+^ monocytes and CD41^–^CD14^+^ monocytes after single-cell RNA-Seq analysis of the PBMCs. (**C**) Selected pathways that were differentially expressed in CD41^+^CD14^+^ monocyte and CD41^–^CD14^+^ monocyte clusters.

To define the biomarkers on the CD41^+^CD14^+^ MPAs, single-cell RNA-Seq analysis was performed on 2 PBMC samples (1 vaccinated and 1 naive). Based on the integrated single-cell gene expression profiles, we combined the 2 samples and projected into uniform manifold approximation projection (UMAP). Using unsupervised clustering analyses and marker mapping, we defined 9 major cell types, including 2 CD14^+^ monocyte subtypes with or without platelets on the surface (CD41^+^ and CD41^–^ monocytes) (Figure 8A). Compared with CD41^–^CD14^+^ subsets, CD41^+^CD14^+^ monocytes had higher expression of platelet-specific markers, such as PPBP and PF4, consistent with the binding of platelets (Figure 8B). The top upregulated DEGs on CD41^+^CD14^+^ monocytes included PLAC8, FCER1A, NRGN, GNG11, CAVIN2, SEPTIN5, JCHINA, and IRF8, while the top downregulated genes included S100A8/9, FN1, VCAN, CFD, DUSP6, C5AR1, IDO, CD14, and SOD2 (Figure 8B and Supplemental Table 4). Particularly, fibronectin 1 (FN1), which interacts with structural and regulatory proteins of HIV-1, including gp41and gp120, was downregulated on MPAs. It has been reported that polymerized (matrix) or degraded (inflammation-associated) FN1 on cells, but not (plasma) dimeric FN1, can enhance HIV-1 infectivity (28–31). The reduced expression of FN1 supported the protective role of MPAs.

Further analysis using the signaling pathways enriched by the DEGs revealed that platelet-CD14^+^ monocytes had higher expression of formation of fibrin clot and FGFR 1c/4 and FGFR3 ligand binding and activation, while lower expression of cytosolic Ca^2+^ levels, alpha-oxidation of phytanate, urea cycle, and interleukin-33 were among the downregulated pathways (Figure 8C and Supplemental Table 5).

## Discussion

SHIV-vaccinated animals demonstrated substantial protection against repeated, IR, low-dose SIVmac251 challenges. To delineate the mechanisms, we found that neither anti-Env antibody nor CD8^+^ cell response was responsible for the reduced viral acquisition. Instead, innate immunity might be the key for preventing viral acquisition. Innate immunity constitutes the first line of immune defense against infections. Innate and adaptive immunity are usually cooperative to combat pathogens. For example, upregulated innate HIV resistance factor APOBEC3G, which was associated with reducing HIV replication and transmission, was accompanied by robust anti-HIV T cell responses (22, 32, 33). Whether and how innate immunity alone prevents HIV transmission has not been fully evaluated and therefore is less appreciated (34). Using this live SHIV vaccine model, we excluded the contribution of adaptive immunity. In searching for innate immunity correlates, we identified an immune tolerance signature characteristic of downregulated IFN/viral response pathways in the protected animals before SIVmac251 challenge. The protected animals also demonstrated higher frequencies of monocyte/T cell platelet aggregates.

For potentially dangerous signals/antigens, the immune system reacts by attacking or remaining unresponsive. Immune tolerance is known as the state of an active, highly regulated unresponsiveness of the immune system to self-antigens or against a particular antigen that can induce an immune response in the body (35). By using “immune tolerance signature” in the manuscript, we specifically referred to the downregulated genes that were involved in the viral/IFN response pathways. IFNs are key players of innate immunity against viral infections. Myeloid cells, such as plasmacytoid dendritic cells and monocytes/macrophages, are the main producers of IFNs after the stimulation of TLRs, cyclic GAMP synthase and IFN-inducible protein 16 (36, 37). IFNs exert their functions though the transcription of numerous anti-HIV IFN-stimulated genes, which include APOBEC3G, TRIM5α, BST2/tetherin, SAMHD1, and MX2 (38). However, type I IFN is a double-aged sword in HIV/SIV infection. The complicated relationship among IFNs and HIV infection, disease progression, and the HIV reservoir is the topic of numerous in-depth reviews published in the past decade (39–43). Type I IFNs, the most predominant and well characterized being IFN-α and IFN-β, have been shown to limit infection and replication of virus both in vitro and in vivo (44–47). Furthermore, administering IFN-α to rhesus macaques has been shown to prevent SIV infection and slow the progression of disease (48). Despite this apparent protective role, sustained stimulation of the immune system by type I IFNs is associated with hyperimmune activation, which further contributes to disease progression (39–41). It is hypothesized that natural hosts of SIV, such as sooty mangabeys and African green monkeys, do not exhibit severe disease because they downregulate IFN-stimulated genes and systemic immune activation during chronic infection (49, 50). Acute SIV infections in these natural hosts are associated with a rapid type I IFN response during early infection but a downregulated IFN response in the chronic infection stages. This contrasts with responses observed during pathogenic SIV infection in rhesus macaques, which exhibit persistently high type I IFN responses (51). These data suggest that the timing and/or duration of the IFN response plays a critical role in disease progression. Our data suggested that downregulated IFN pathways were associated with protection. This might be due to the low immune activation in the animals that had fewer IFN pathways activated. As elevated immune activation not only enhances HIV-1 pathogenesis but also is a high-risk factor for HIV acquisition and transmission (52–55), low IFN pathway activation might be favorable for preventing SIV acquisition. Our data were also consistent with the finding that HIV-exposed seronegative individuals had reduced expression of interferon regulatory factor 1 (52, 56). Both type I and type II IFNs are major players in the immune responses against virus (57). However, the responses must be properly regulated to minimize the tissue damage (58). In pathological conditions, both IFNs contribute to the immunopathology of autoimmune diseases (59–62). Type I IFNs are thought to affect innate autoimmune responses, as they are produced during the early stages of the innate immune responses, while IFN-γ, the only member of the type II IFN family, is pivotal to adaptive autoimmune responses, as its function is to promote T cell differentiation and B cell immunoglobulin class switching (59–61). Given the significant overlap of genes driven by types I and II IFNs, both play a pivotal role in the development and severity of IFN-related autoimmune diseases (59).

While human NK cells express variable levels of CD8α, rhesus macaque NK cells are uniformly CD8αα^bright^ (63). This leads to the complication that the depletion strategies utilizing anti-CD8α antibody, for example M-T807R1 used in this study, will remove both CD8^+^ T cells and NK cells as well. Based on the result of the CD8 antibody depletion SIV challenge experiment, at least in this study neither CD8^+^ T cells, nor NK cells, played important roles in preventing SIV acquisition. Therefore, we focus mainly on myeloid cells in this study. However, in other settings, NK cells might contribute to preventing viral acquisition. NK cells provide direct killing of infected cells via the release of perforin- and granzyme-containing granules. NK cells are also major producers of type II IFN, TNF, and GM-CSF. The role and importance of NK cells in HIV/SIV pathology are still quickly evolving areas of research. SIV infection was found to increase perforin expression and cytotoxicity in CD16^+^ NK cells in rhesus macaques (64); however, other studies describe a negative effect on NK cells (65, 66). It has also been demonstrated that NK cell memory can be induced in primates following both SIV infection and vaccination (67). In fact, both NK cells and myeloid cells can exhibit epigenetic and functional reprogramming (i.e., trained innate immunity) (68).

Platelets are small non-nucleated cell fragments (from megakaryocytes) that play crucial roles in managing vascular integrity and regulating hemostasis (69). In addition to hemostasis, in response to platelet or leukocyte activation, platelets can form platelet-leukocyte aggregates in the peripheral blood, which contribute to immune defense against viral infections. PF4 (also known as CXCL4), a small chemokine released from the α-granules inside the platelet upon activation, has broad-spectrum inhibitory activity against HIV-1 (70–72). It is known that PSGL-1 on activated monocytes and T cells can bind CD62P, which is P selectin, on activated platelets, to form platelet-monocyte aggregates. The presence of platelets bound to monocytes can be detected by flow cytometry using the platelet marker CD41, also known as integrin alpha 2b. CD62P shows platelet activation, but if PF4 is also stained in the aggregates, it implies activation with PF4 secretion, and such activated platelet-monocyte aggregates would make high local levels of protective PF4. Platelets can endocytose and process virions including HIV, and the incubation of pseudo-HIV virions with platelets led to granule secretion and platelet-leukocyte aggregate formation (73). Platelets and MPAs have multifarious roles in HIV-1 infection. During pathological HIV-1 infection, they were enhanced and correlated with circulating monocytes, viremia, markers of immune activation, and disease progression (74–76). However, in this nonpathological SHIV vaccine model, we found that monocyte- or T cell–platelet aggregates may confer protection against pathological SIV acquisition. Platelets can combat HIV through multiple mechanisms: releasing antiviral molecules, phagocytosing viral pathogens, and producing reactive oxygen species. Early studies in an ALVAC HIV vaccine showed that CD14^+^ monocytes were associated with improved efficacy, but the protective mechanisms remain elusive (23, 24, 77). Here, we found that PF4-expressing CD14^+^CD16^–^ monocyte– and CD4^+^ T cell–platelet aggregates exhibited strong correlations with protection, suggesting that PF4 might be involved in preventing or aborting early viral SIV/HIV replication. The PF4 released by platelets bound in aggregates with monocytes would be more effective than circulating PF4 because of the higher local concentration. Platelets can also release RANTES, which binds to CCR5 and blocks HIV/SIV infection. Based on our data, we speculate that thrombocytopenia in humans would lead to high HIV transmission. In agreement with this, a recent study found that low platelet count was independently correlated with an increased risk of infection in patients with primary immune thrombocytopenia (78).

The animals were infected with SHIV 18 months before the SIV challenge, and the SHIV had been long cleared from the blood. However, it is known that reservoirs of SHIV can persist and reemerge, as we saw after CD8 depletion. The durability of the protective innate immunity may depend on a persistent viral reservoir. The challenge for translating innate mechanisms, such as the platelet PF4, is to identify strategies to activate these mechanisms without the need for a live viral vaccine. Such mechanisms could involve inducing epigenetic changes in myeloid cells, such as those described as mediating trained innate immunity (79, 80). Note that the platelet-monocyte aggregates involve a changed activation state of these myeloid cells, which could be induced by other mechanisms.

Although this study demonstrates a surprising role of innate immune responses as correlates of protection, the limitations of the study, which include the small sample size, using only 1 animal/group for the single-cell sequencing, and potential complications associated with reuse of animals from prior studies, need to be kept in mind. Even if the main conclusions were confirmed using other methods such as qPCR and flow cytometry, in the future, more rigorous, and confirmatory, mechanistic studies would be required to validate the descriptive and correlative results of this study. One caveat of this study is that the transcriptional response analysis was done only on PBMC samples, and the response in rectal tissues (prior to challenge), which may have unique mucosa-related characteristics, was not able to be investigated. Indeed, an ongoing transcriptome analysis of the rectal mucosal tissues from another HIV vaccine study (7), where, like this study, anti-Env antibody did not play a protective role, revealed the upregulation of signaling by retinoic acid (Sui et al., unpublished data). Nevertheless, we found that 1) there were 2 times more downregulated DEGs than upregulated ones after vaccination; 2) bleeding/coagulation-related pathways were altered, and 3) the C3 complement gene was among the top 10 upregulated genes. These findings are consistent with those from the circulation, suggesting common mechanisms. Another limitation is that the findings of our study do not directly lead to a new vaccine in humans; due to safety reasons, an attenuated live HIV vaccine will never be used in humans. However, the described mechanisms of protection might be leveraged for vaccine development in the future.

Our data demonstrated that innate immunity was responsible for the reduced risk of SIV viral acquisition in the absence of anti-Env antibody and CD8^+^ cell responses in an SHIV-vaccinated and SIV challenge macaque model. Specifically, upregulated monocyte-/T cell–platelet aggregates and downregulated IFN pathways were correlated with protection. Further investigation of innate immunity, and learning to exploit platelet-mediated protection, may pave new avenues for the development of novel HIV vaccines or therapeutic strategies.

## Methods

### Sex as a biological variable.

Our study examined male and female animals, and similar findings are reported for both sexes.

### Animals.

Eighteen adult Indian rhesus macaques (*Macaca mulatta*) were housed in the NIH animal facility. Before the study, the animals were negative for SIV; Macacine herpesvirus 1; simian retroviruses 1, 2, and 5; and simian T cell leukemia/lymphotropic virus type 1. The information on sex, age, weight, and MHC alleles of these animals is shown in Supplemental Table 1.

### SHIV vaccination.

Twelve macaques were equally distributed into 2 groups. While 6 of the macaques were naive (defined as naive-SHIV group), the other 6 macaques had received HIV mucosal vaccine as described before (defined as vac-SHIV group). Briefly, the animals were primed with MVA plus adjuvant (TLR agonist and IL-15) IR along with rhesus full-length single chain gp120-CD4 fusion protein (rhFLSC) plus mutant *E*. *coli* lymphotoxin (mLT) in oral nanoparticles at weeks 0 and 4, followed by boosting with rhFLSC plus mLT in oral nanoparticles at weeks 8 and 12. At week 20, all 12 animals were challenged with multiple low doses of IR inoculation of (1:35 diluted) SHIV_SF162P4_ stock (M661-derived harvest 1 dated October 5, 2006), which was provided by Nancy Miller, National Institute of Allergy and Infectious Diseases (NIAID), NIH, until the animals were confirmed infected. SHIV RNA levels determined by nucleic acid sequence–based amplification (NASBA) assays were monitored by Advanced BioScience Laboratories. About 3 months after the confirmation of infection, all 12 animals controlled their VLs, including the initially naive animals. No anti-SIV Env antibody was detected in these 12 animals.

### SIVmac251 challenges.

Eighteen months later, all 12 SHIV-vaccinated animals plus 6 additional naive animals were subjected to 8 weekly low-dose SIVmac251 challenges. The SIVmac251 challenge stock (swarm of tier 1 and tier 2), SIVmac251 “Desrosiers” 2010-Day8, lot 305342b, was provided by Nancy Miller, NIAID, NIH. The titers of the stock virus were 2.5 × 10^5^ tissue culture ID_50_ /mL in C8166-SEAP cells and 1 × 10^5^ tissue culture ID_50_/mL in rhesus 221 cells (prepared at Quality Biological). The stock virus was diluted at 1:1,000 and was IR administered to the animals as described before (7, 81). The VL was determined by NASBA measurements on Gag (Advanced BioScience Laboratories). The cutoff threshold for VL measurement was 10 or 50 copies/mL.

### Viral envelope sequencing.

Single genome amplification and Sanger sequencing was performed on plasma samples from all infected macaques. The entire Env gene was sequenced using a limiting-dilution PCR to ensure that only 1 amplifiable molecule was present in each reaction mixture. Viral RNA was isolated using the QIAamp Viral RNA Mini Kit (QIAGEN) and immediately reverse-transcribed into single-stranded cDNA using SuperScript III (Invitrogen) with the Env-specific primer SIVEnvR1 5′-TGTAATAAATCCCTTCCAGTCCCCCC-3′. cDNA was serially diluted and distributed among independent PCRs to identify a dilution where positive wells were less than 30% of the total number of reactions. PCR amplification was performed with Platinum Taq High Fidelity polymerase (Invitrogen) in a 20 μL reaction. First-round PCR was performed with sense primer SIVEnvF1 5′-CCTCCCCCTCCAGGACTAGC-3′ and antisense primer SIVEnvR1 5′-TGTAATAAATCCCTTCCAGTCCCCCC-3′. Next, 1 μL of the first-round PCR product was added to a second-round PCR that included the sense primer SIVEnvF2 5′-TATAATAGACATGGAGACACCCTTGAGGGAGC-3′ and antisense primer SIVEnvR2 5′-ATGAGACATRTCTATTGCCAATTTGTA-3. Correct-sized amplicons were identified by agarose gel electrophoresis and directly sequenced.

### ELISA to detect SIV gp120-specific antibody responses.

The antigen-specific binding assays were performed similarly as previously described (82, 83). A total of 100 ng/well of the SIVmac251 gp120 protein (*ABL*) was used as the coating antigen followed by 2% sodium casein for blocking. Serum samples were applied in duplicate with a series of 4-fold dilutions starting from a 1:150 dilution, and the plates were incubated at room temperature for 1 hour. After 4 washes, 1:20,000 dilutions of Goat anti-Monkey IgG (H+L) Secondary Antibody [HRP] (Novus Biologicals) and TMB substrate were added as described (82, 83). OD_450_ was measured after quenching with 1 M H_3_PO_4_ solution.

### CD8 depletion study.

Two CD8 depletion studies were conducted using MT807R1 antibody as shown before (84). Briefly, in the first pilot study, CD8 depletion was performed on 6 animals as shown in Figure 3A. We collected blood and rectal pinch samples to characterize the CD8^+^ cell kinetics in rectal mucosa and blood. In the second study, 1 depletion group (*n* = 3) and 1 nondepletion group (*n* = 3) were subjected to CD8 depletion as shown in Figure 4A. The animals were challenged by SIVmac251 at days 7, 10, 14, and 17 after CD8 antibody treatment while the CD8^+^ T cell levels were still undetectable. VL measurement and deep sequencing of the viral envelope were run to identify SIV and SHIV.

### Flow cytometric analysis of virus-specific T cell responses in PBMCs and colorectal tissues and MPAs in PBMCs.

Intracellular cytokine analysis and CM9-dextramer staining were used to measure the virus-specific T cells in PBMCs and mononuclear cells isolated from colorectal lamina propria by flow cytometric analysis, as previously described in detail (22, 85). Mamu-A*01–positive CM9-dextramer was obtained from ImmuDex. The details of collecting and processing of the colorectal tissues were described before (22, 55, 86). For APC-labeling anti-PF4 antibody, Lightning-Link kit (Novus Biologicals) was used per the manufacturer’s instructions. For staining MPAs, antibody mixture was added to the PBMCs, which were thawed and washed. After incubation for 30 minutes, yellow viability dye was added for 10 minutes of incubation. Following washing, the cells were fixed, and data were acquired with a BD LSR II flow cytometer. The antibody information is listed in Supplemental Table 6. FlowJo software (Tree Star Inc) was used for data analysis.

### Viral neutralization assays.

SIV viral neutralization assays were performed as described before (87). Briefly, plasma samples from all samples were assayed against the tier 1 SF162.LS immunogen strain and against a tier 1 and a tier 2 clone of SIVmac251 in TZM-bl cells (obtained from the NIH AIDS Research and Reference Reagent Program, catalog 8129) using MLV-pseudotyped virus as a negative control for nonspecific inhibition of signal in the assay. MLV and the tier 2 SIVmac251.41 were assayed starting at a 1:20 dilution and the 2 tier 1 viruses at a starting dilution of 1:30 to reach an endpoint titer for all samples. No background activity against MLV was detected in serum samples. The serum dilution at which RLUs were reduced 50% compared with virus control wells (no test sample) was calculated for each sample.

### ADCC assay.

We tested the samples using the Luciferase-based ADCC assay against the SIVmac251 and HIV-1 SF162 IMC-infected target cells, adapting previous methodology to derive HIV-1 IMC-infected target cells (88). This is an ecto-IMC generated using the HIV-1 NL4-3 backbone with the insertion of the SIVmac251 or the HIV-1 SF162 envelopes and the Luciferase reporter genes (provided by C. Ochsenbauer, University of Alabama, Birmingham, Alabama, USA) (89). The analysis of the results was conducted after subtracting the background detected with the preimmunization samples. After background subtraction, results would be considered positive if the percentage of specific killing is above 10%. The magnitude of the responses is reported as AUC that was calculated from dilution curves using a nonlinear trapezoidal method (90).

### ADCP assay.

Gp130 SIVmac251 protein–coupled fluorescent beads (1.0 μm Fluorospheres NeutrAvidin-labeled microspheres, Thermo Fisher Scientific) were added to diluted serum samples (duplicated, 1:50 dilution in 0.1%/BSA/PBS) or antibody controls (including purified IgG from SIV-infected rhesus macaque; 50 μg/mL, anti-HIV immunoglobulin; 1 mg/mL, influenza receptor binding site-specific broadly neutralizing monoclonal antibody; 50 μg/mL) to form immune complexes by incubation for 2 hours at 37°C/5% CO_2_. THP-1 cells (American Type Culture Collection) were resuspended at 10 million cells/mL in RPMI without serum and treated with anti-human CD4 (20 μL/mL) for 15 minutes at 4°C. Treated THP-1 cells (at 0.25 million cells/mL in THP-1 media: RPMI + 10% FBS + 1% penicillin/streptomycin) were added to immune complexes and spinoculated at 4°C, 1,200*g*, for 1 hour, then incubated at 37°C/5% CO_2_. Cells were fixed by adding 4% paraformaldehyde and analyzed by flow cytometry. ADCP scores were calculated: mean fluorescence intensity × percentage of bead-positive THP-1 cells for test serum or mAb divided by the no-antibody control. Positivity criteria were 1) cutoff = 95th percentile of study-, antigen-, and experiment-specific baseline ADCP scores and at least 1; 2) sample considered positive when postbaseline ADCP score ≥ cutoff and 3 times over antigen- and experiment-specific paired baseline ADCP score.

### RNA-Seq experiment.

PBMC samples were collected before SIVmac251 challenge (1 or 2 months before SIVmac251 challenges). The cells were diluted to 2 × 10^6^/mL to 4 × 10^6^/mL. The O samples were spun down and put into TRIzol (Invitrogen) for RNA isolation. PBMCs were added to 6-well plates in R10. To enrich for myeloid cells, the M samples were washed with warm PBS (3 times) after 4 hours of incubation to remove the suspension cells. Then both P and M samples were incubated with SIVmac251 (1:100 dilution) for 18 hours. After centrifugation (300*g*, 5 minutes, room temperature) to remove the supernatant, TRIzol was added, followed by using QIAGEN RNA isolation kits to isolate RNA. The RNA samples were then subjected to the RNA-Seq experiment. The RNA-Seq library was constructed using TruSeq Stranded mRNA kit (Illumina).

### qPCR experiment.

RNA samples from M samples were used for the qPCR experiment. For measuring Gag, GAPDH, and the 22 genes listed in Figure 6, TaqMan probe and primer sets were used (Thermo Fisher Scientific; Supplemental Table 5). RNA transcription and qPCR were performed per Bioline USA Inc instructions. Comparative threshold cycle method of relative quantification (PerkinElmer User Bulletin no. 2) was used to calculate relative mRNA expression levels as described previously (91, 92). After normalization to GAPDH, each gene expression in 1 animal was used as the reference to calculate that gene’s fold-changes in the rest of the animals.

### RNA-Seq analysis.

RNA sequencing, alignment, and quantitation were performed by Macrogen. Downstream analysis and visualization were performed within the NIH Integrated Analysis Platform using R programs developed by a team of National Cancer Institute bioinformaticians on the Foundry platform (Palantir Technologies). Briefly, quality of raw sequences was checked using FASTQC v.0.11.7, and then reads were trimmed using Trimmomatic v 0.38. Reads were aligned to the *Macaca mulatta* reference genome (GCF_000772875.2_Mmul_8.0.1). StringTie v.1.3.4d was used to quantitate raw expression from aligned reads. These gene counts were then imported into the NIDAP platform, where genes were filtered for low counts (<1 count per million) and normalized by quantile normalization using the limma package (93). DEGs were calculated with limma-Voom. GSEA was performed using the fgsea package (94), and further pathway enrichment was performed using Fisher’s exact test (l2p, https://github.com/ccbr/l2p; commit ID a3878df).

### Single-cell RNA-Seq analysis.

Two PBMC samples (VS5 and N2) were subjected to 10x Genomics single-cell RNA-Seq. The viability of samples was confirmed to be more than 90% prior to further processing. About 10,000 cells from each sample were loaded with the goal of capturing 6,000 cells. 10x Genomics 3′ v3.1 single-cell gene expression libraries were prepared as instructed by the 10x Genomics user’s guides. The cDNA libraries were sequenced on the Illumina NextSeq 2000 with a target depth of approximately 50,000 reads per cell. Sequencing read structure was as follow: 28 bp (Read1, cell barcode, and unique molecular identifier), 8 bp (sample index), 91 bp (Read2, library insert). For biocomputational analysis, base calling was performed using RTA 3.9.2, and demultiplexing was performed using Cell Ranger v6.1.1 (Bcl2fastq 2.20.0). Alignment was performed using Cell Ranger v6.1.1 (STAR 2.7.2a). Sequenced reads were aligned to custom macaque reference (Enhanced_Macaca_mulatta_10.104, modified macaque reference and annotations courtesy of Stefan Cordes, National Heart, Lung, and Blood Institute, NIH, Bethesda, Maryland, USA). The 10x Genomics Cell Ranger pipeline (v6.1.1) was applied to align reads to the reference with default settings and to generate a gene expression matrix. Cells with low (<500 genes) and more than 10% mitochondrial reads were removed from the rest of analysis. Standard workflow in Seurat _4.0.4 was used for normalization (log normalization with scale factor 10,000), scaling, linear and nonlinear dimensional reduction, and clustering (95). Clustering resolution of 0.5 was used and 13 different clusters were generated. Differential analysis was performed using Seurat_4.0.4 using MAST method (96). ReactomeGSA package was used for pathway analysis (https://github.com/reactome/ReactomeGSA; commit ID d36886e). Cell annotation was performed using cell-specific markers. Annotated clusters were CD19+ Bcells, CD3E+CD8A+ Tcells, CD3E+CD4+ Tcells, CD3E+ Tcells, CD16+CD8A+ NKcells, CD14+ Monocytes, CD14+ Monocytes & CD41+Platelets, CD16+ activated Monocyte & NKcells (Natural killers), and Proliferative cells.

### Statistics.

We performed statistical analyses with Prism version 9 (GraphPad). Mann-Whitney and Wilcoxon tests were used as shown in the figures. Spearman’s analyses were used for correlations. A 2-sided significance level of 0.05 was used for all analyses.

### Study approval.

The animal facility is part of the NIH animal program that is fully accredited by Association for Assessment and Accreditation of Laboratory Animals International and has an active NIH Office of Laboratory Animal Welfare assurance (Animal Welfare Assurance Number D16-00602). All animal care adheres to the Animal Welfare Act and follows standards proposed by the *Guide for the Care and Use of Laboratory Animals* (2011, National Academies Press). All work involving animals was conducted under an animal protocol that was approved by the National Cancer Institute (NIH) Animal Care and Use Committee.

### Data availability.

The raw data for RNA-Seq analysis and single-cell RNA-Seq have been deposited in NCBI’s Gene Expression Omnibus (GEO) and are accessible through GEO Series accessions GSE241963 and GSE242726, respectively. The code used to produce some of the bulk RNA-Seq results shown in this manuscript is available at https://github.com/NIDAP-Community/Protection-against-intrarectal-SIV-by-a-SHIV-vaccine (commit ID 78c7e20). The envelope sequence data are accessible though BankIt2743662: OR571932 - OR571984; BankIt2744750: OR571985 - OR572016; BankIt2744765: OR572017 - OR572091; and BankIt2744770: OR572092 - OR572098.

## Author contributions

YS, CJM, and JAB conceived and designed the projects. TJM and MC did the bulk RNA-Seq analysis; CMF and BFK performed viral envelope sequencing; KD and MCK did single-cell analysis; YS, CM, and TFG performed and interpreted the platelet experiments; CCL, DM, GF, LDW, XS, and GDT ran viral neutralization and ADCC and ADCP studies; MWB, JAK, and their team performed the animal studies; YS processed blood and tissue samples, prepared bulk and single-cell RNA-Seq samples, and ran qPCR and cellular assays; JL and SEH ran anti-SIV Env ELISA experiments; and YS and JAB wrote the manuscript with input from all the coauthors. All authors participated in interpreting the data.

## Supplementary Material

Supplemental data

Supplemental table 2

Supplemental table 4

Supplemental table 5

Supporting data values

## Version 1 06/24/2024

Electronic publication
