# Supplementary material for: Innate protection against intrarectal SIV acquisition by a live SHIV vaccine
Source: JCI Insight. 2024 May 21;9(12):e175800. doi: 10.1172/jci.insight.175800 (PMC11383375; doi:10.1172/jci.insight.175800)
Supplement: Supplemental data [file jciinsight-9-175800-s021.pdf]

Supplementary Figure 1: The MDSCs of the SHIVSF162P4-vaccinated animals were assessed in the PBMC samples collected one month before SIVmac251 challenges. PBMCs from three naïve animals were included as control.

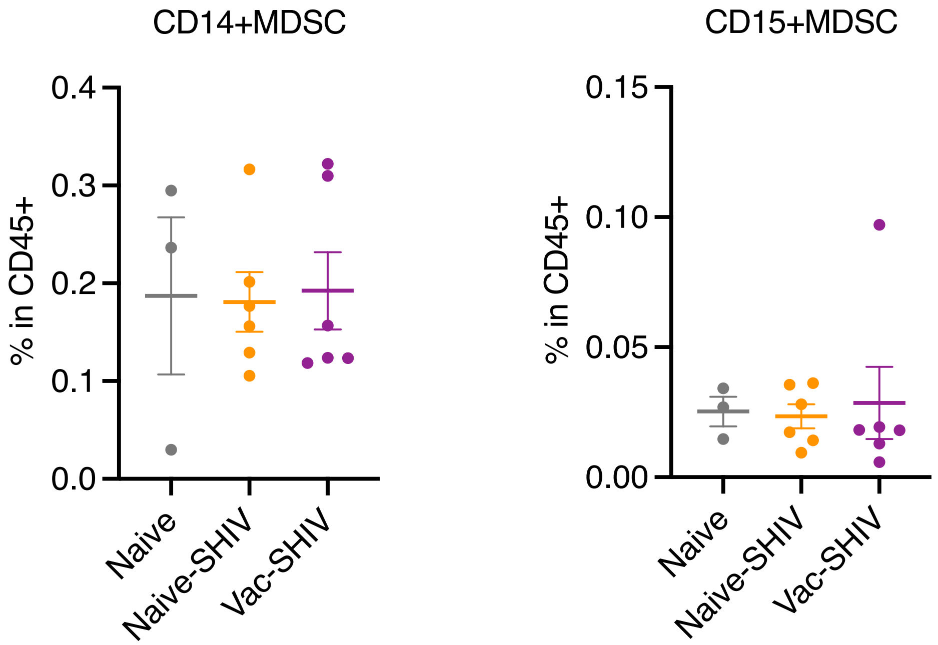

Supplementary Figure 2. The CD8+ and CD4+ cells in peripheral and rectal mucosa, as well as plasma viral loads after administration of an anti-CD8 alpha antibody MT807R1. A). Schematic diagrams of CD8 depletion using MT807R1 antibody. B). The dynamics of CD8+ and CD4+T cells in peripheral blood (D) after the administration of anti-CD8 alpha antibody. C). The plasma viral loads in the six CD8-depleted animals from either Naïve-SHIV group or Vac-SHIV group. D). The dynamics of CD8+ and CD4+T cells in rectal pinches after the administration of anti-CD8 alpha antibody.

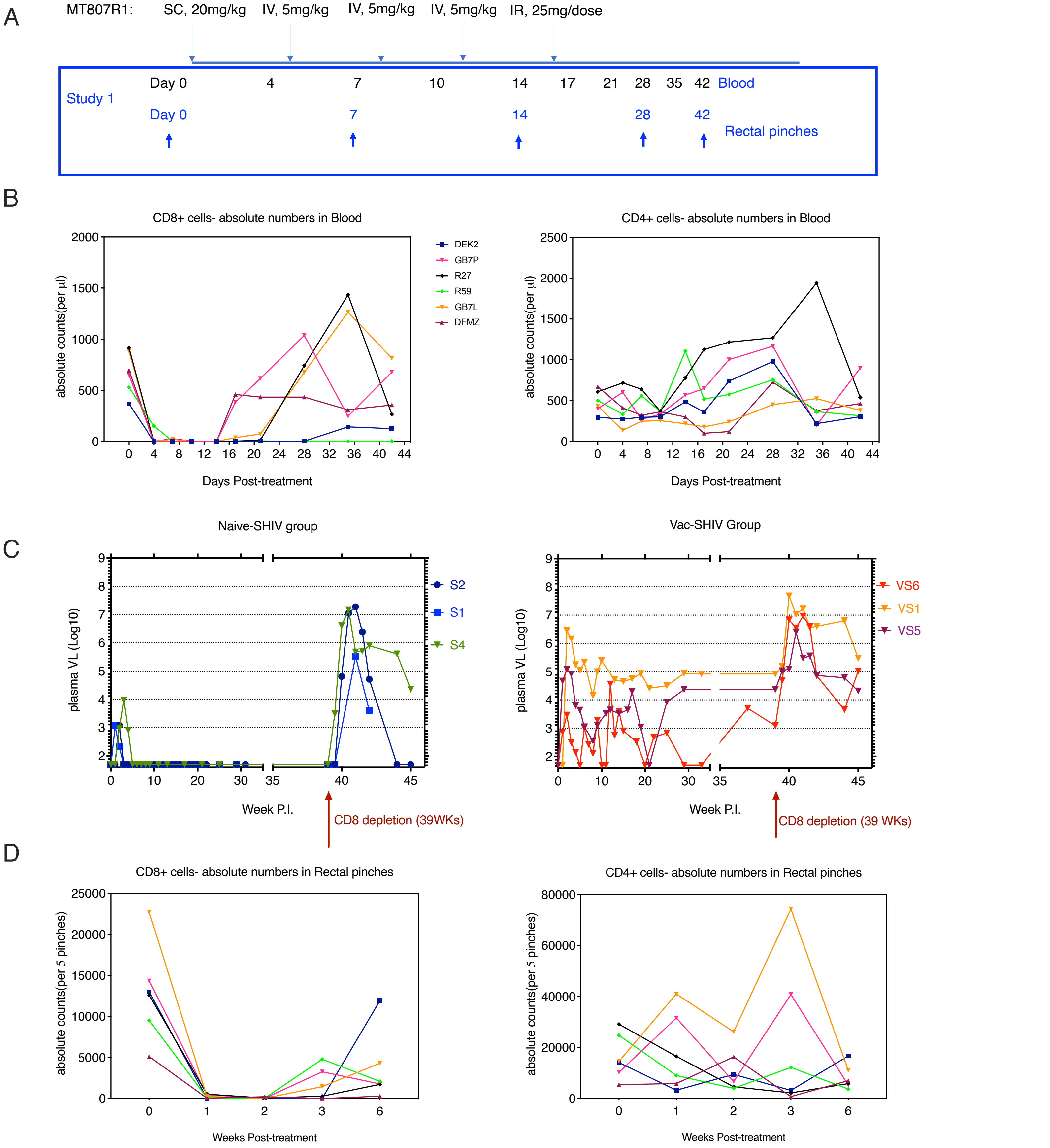

Supplementary Figure 3. The DEGs between vac-protected vs vac-infected animals in different sample types (samples were taken before SIVmac251 challenges). O samples: original PBMC without any stimulation; P samples: PBMC incubated/stimulated with SIVmac251 virus for 18 hours; M samples: enriched myeloid cells incubated/stimulated with SIVmac251 virus for 18 hours. A). PCA plot of PBMC and myeloid cells after exposed to SIVmac251 virus for 18 hours (O and M samples). B). Venn Diagram of DEGs between vac-protected vs vac-infected animals in three types of samples. C). Volcano plots of the three types of samples.

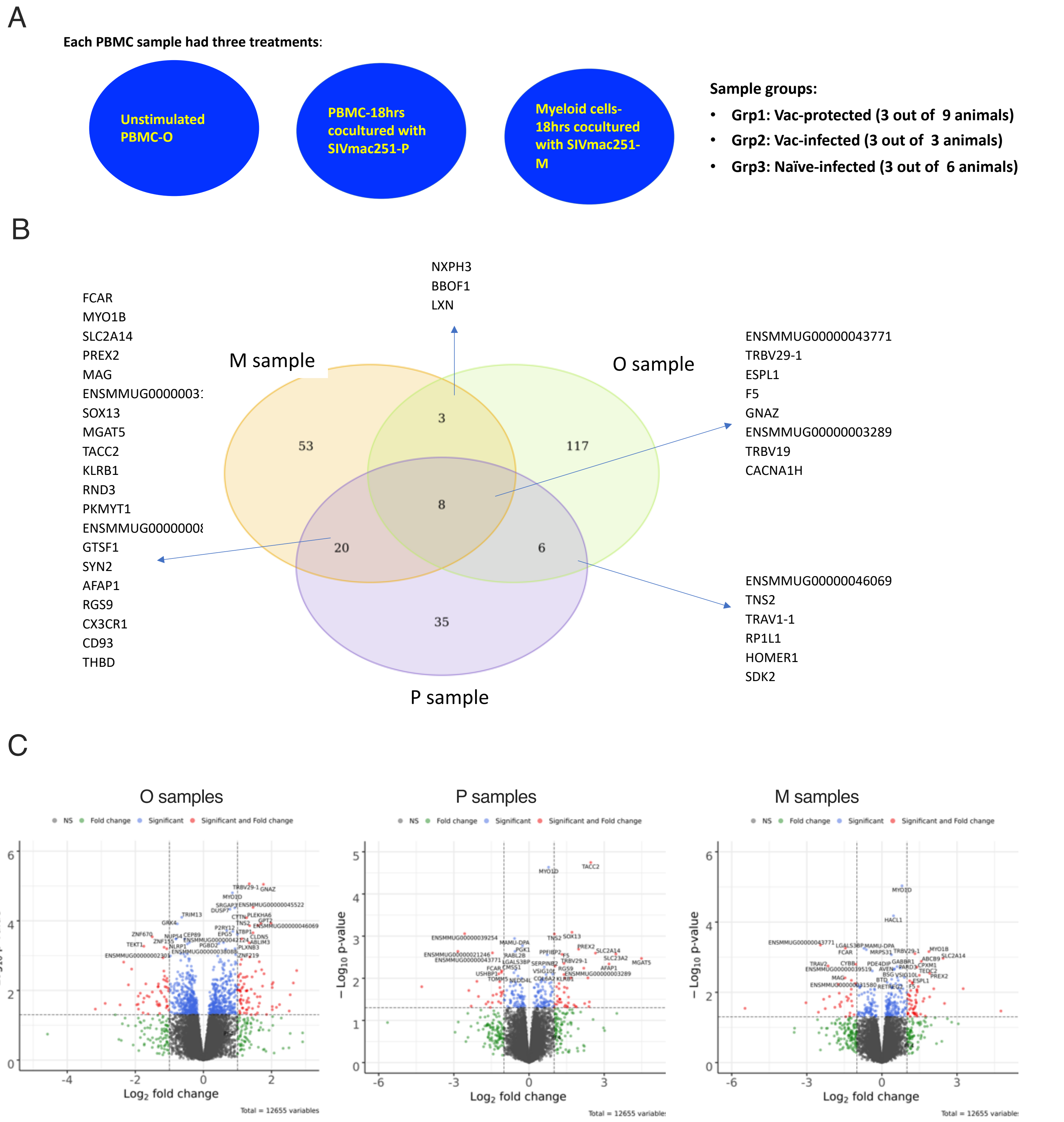

Supplementary Figure 4. The immune tolerance gene signature of the SIV-exposed myeloid cells in naive and SHIV vaccinated animals. The M (myeloid cell enriched) samples were incubated with SIVmac251 for 18 hours. RNA was isolated. q-PCR was to measure the RNA expression levels of the genes. These genes were involved in the following pathways: interferon alpha/beta/gamma signaling; Response to LPS, cytokines; Responses to virus, Influenza A. Two upregulated genes, SPP1 and PDGFA, were included as technical controls. Mann-Whitney was used for comparison. Vacc: SHIV vaccinted animals (n=12) vs. Naive: Naive animals (n=6)

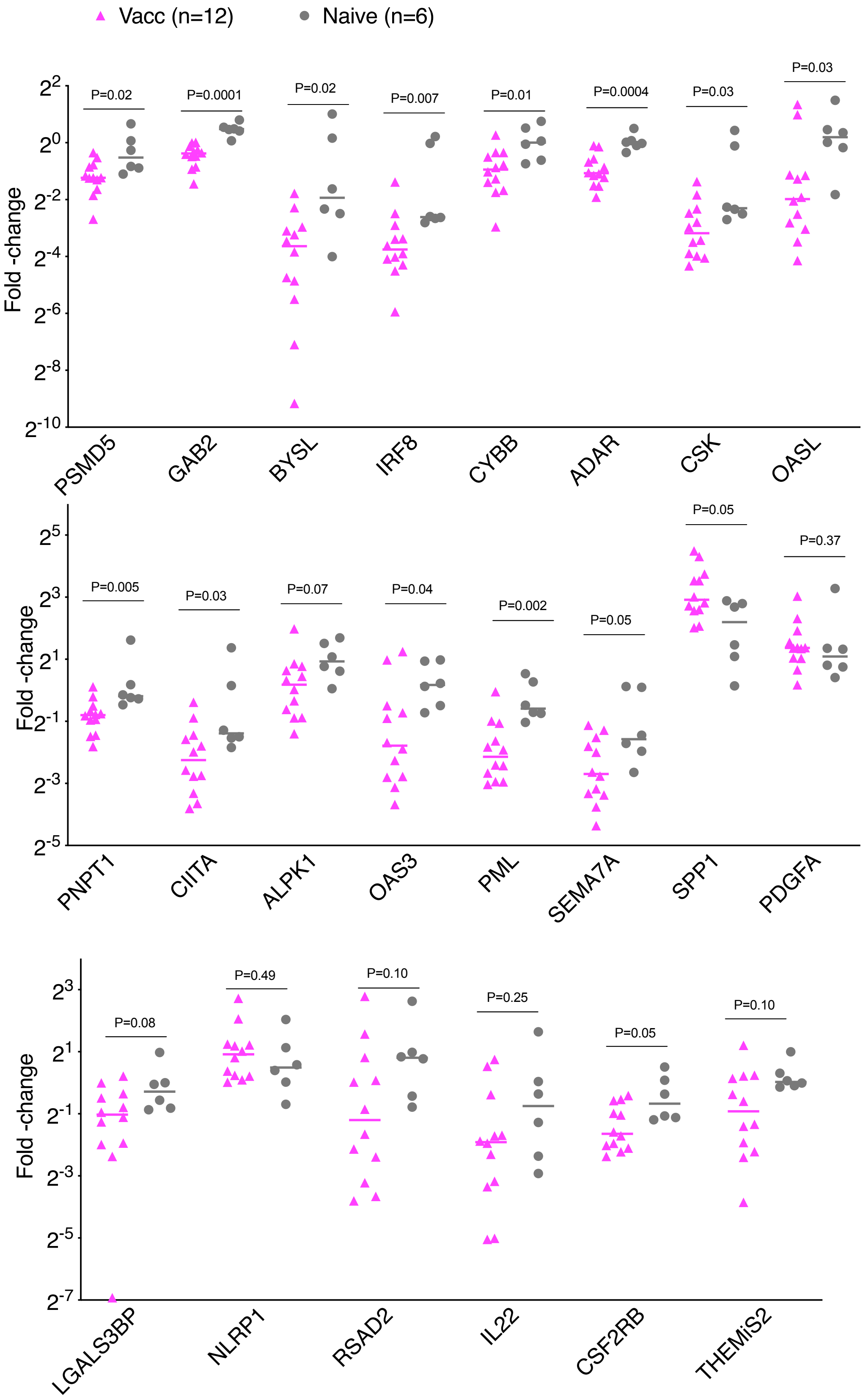

Supplementary Figure 5. The comparison between genes in the Mamu A*01 positive and negative animals that have been vaccinated by SHIV vaccine. q-PCR was used for measuring the relative expression level of the down- and upregulated- DEGs between the protected and infected animals (n=12). These genes were involved in the following pathways: interferon alpha/beta/gamma signaling; Response to LPS, cytokines; Responses to virus, Influenza A. Two upregulated genes, SPP1 and PDGFA, were included as technical controls. Mann-Whitney was used for comparison.

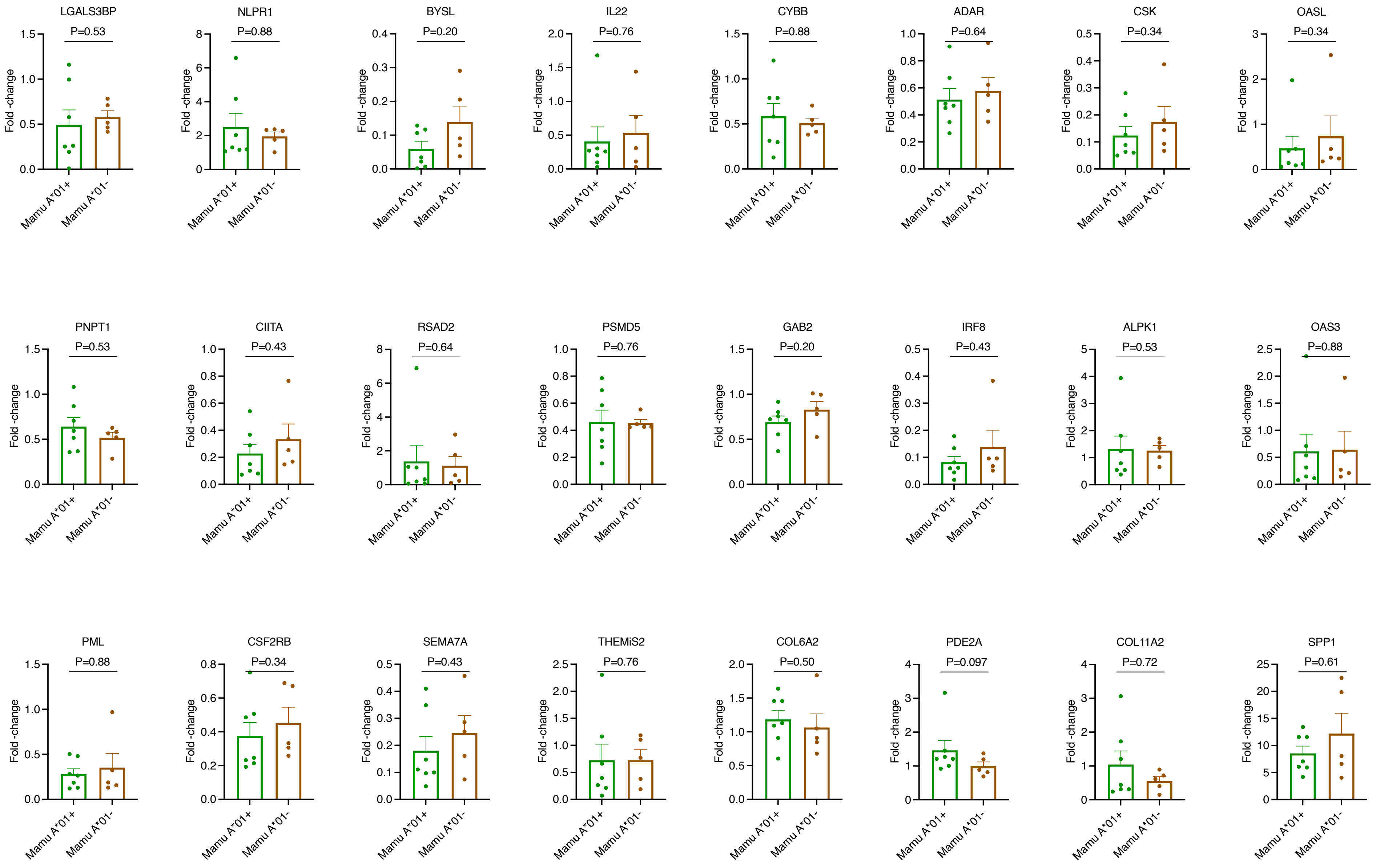

Supplementary Figure 6. The gating strategy for platelet-leukocyte aggregates. PBMC samples were collected before SIVmac251 challenges. After incubation with antibody mixture, the cells were washed and fixed before data acquiring using LSRII. Gating strategies are shown. Color plates are the plots from the sample with all the antibody mix, while the black contour plots are the Fluorescence Minus One (FMO) control. FMO controls and the fully stained sample are not from the same animals.

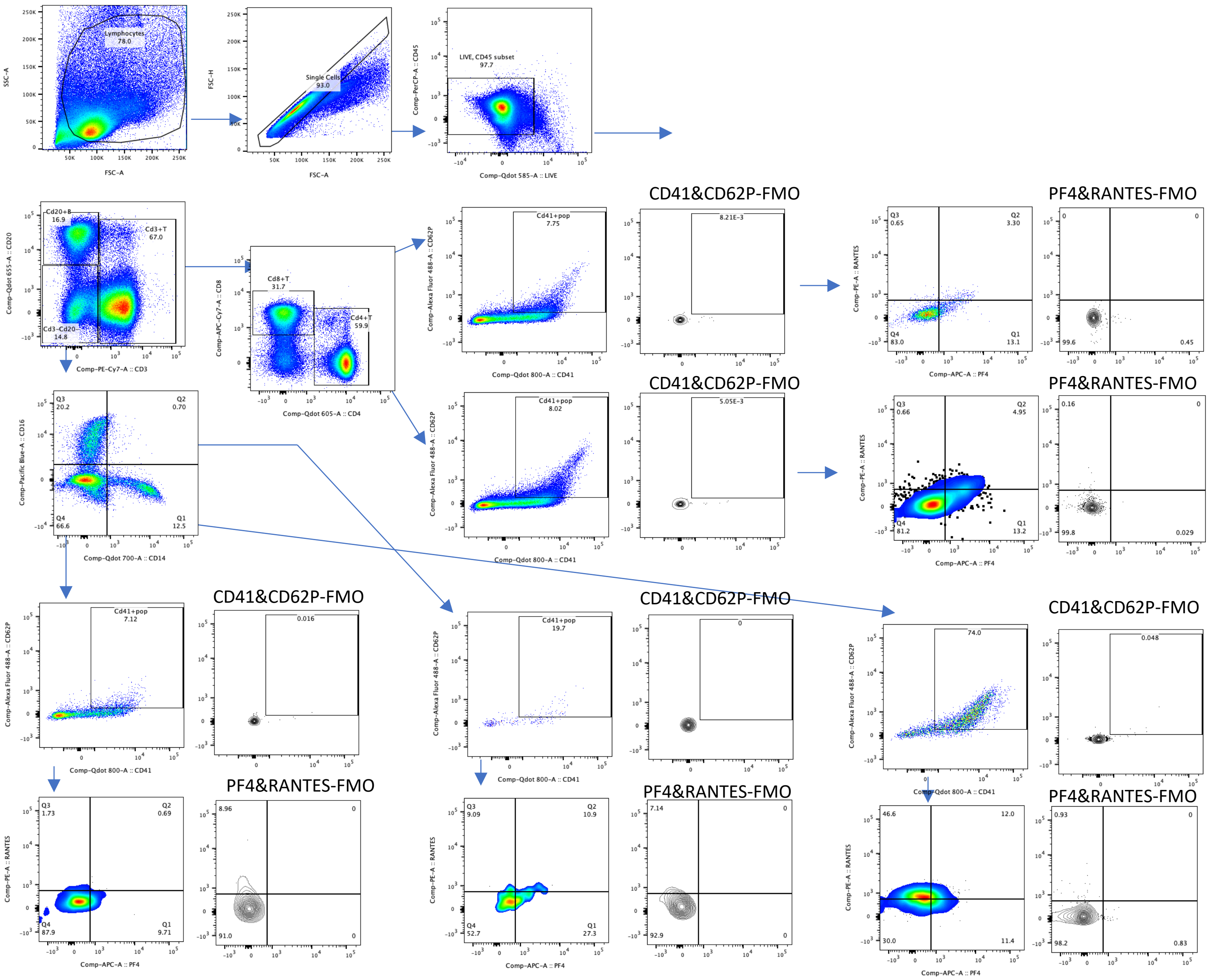

Supplementary Figure 7. The frequencies of activated platelet and PF4 on monocytes and CD4+T cells in the Mamu A*01 positive animals were compared to those of Mamu A*01 negative animals. 12 SHIV vaccinate animals were included, and Mann-Whitney analysis were used for comparisons.

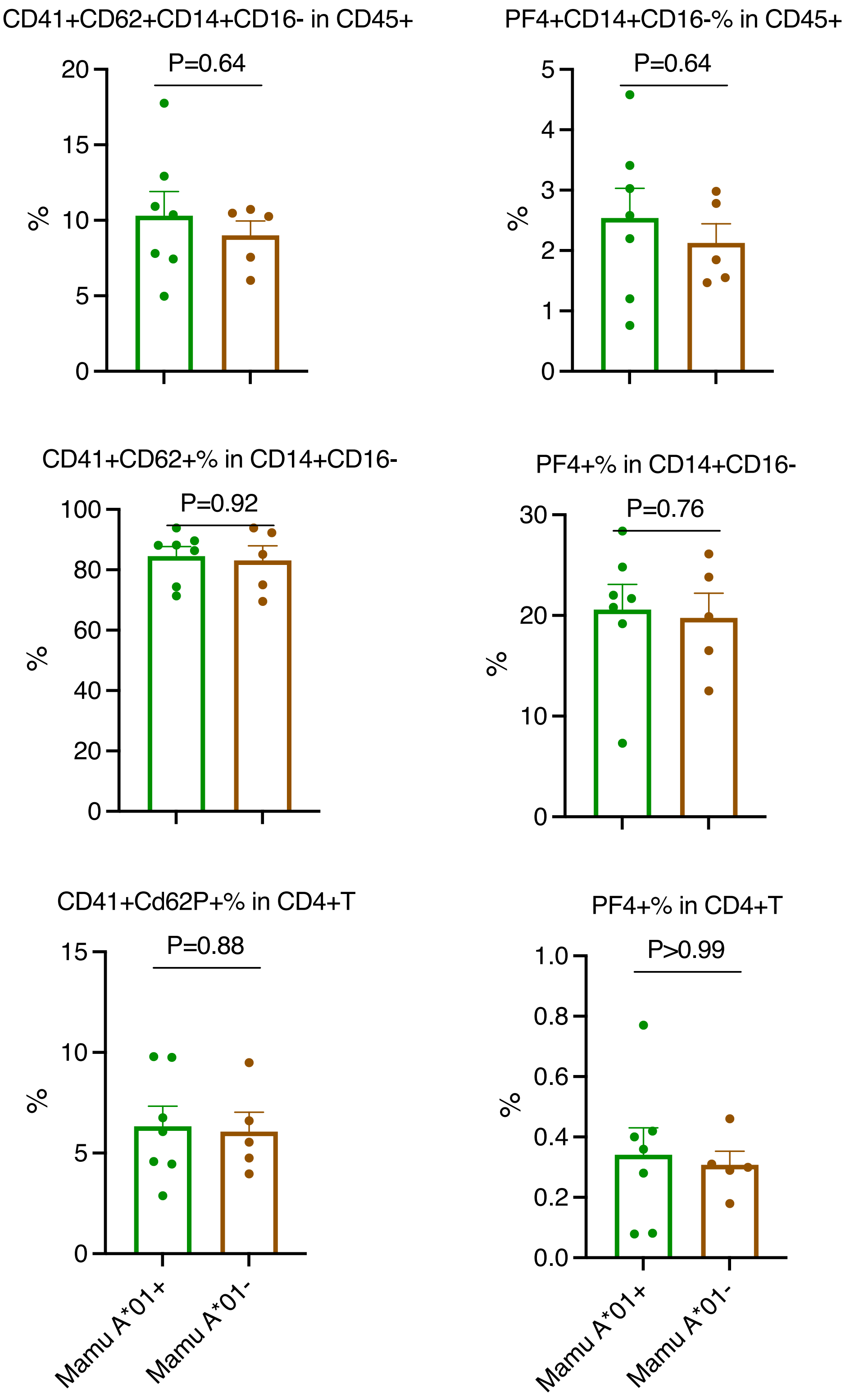

Supplementary Table 1. Animal information

Animal -ID	Group	Aniaml name	Sex	Date of birth	Weight (kg)	A01	B08	B17	B29	Outcome after SIV Challenges	SIV challenge outcome	SIV Challenge outcome MHC A01+	SIV Challenge outcome MHC A01-	1st CD8 Depletion	2nd CD8 depletion (Depletion grp in red)	RNAseq
VS1		GB7L	F	7/28/10	4.3	-	-	-	-	SIV	3		3	GB7L-VS1		GB7L-VS1
VS2		DEK3	M	3/30/10	5.1	+	-	-		No VL	8	8			DEK3-VS2	DEK3-VS2
VS3	Vac- SHIV	825/MG6	F	1/1/10	5.8	-	-	-	-	No VL	8		8		825-VS3	
VS4		DX8T	F	5/19/10	4.1	-	-	-	-	No VL	8		8		DX8T-VS4	
VS5		DFMZ	M	1/1/11	3.2	+	-	-	-	SIV	5	5		DFMZ-VS5		DFMZ-VS5
VS6		GB7P	F	5/31/10	4.5	-	-	-	-	SHIV	8		8	GB7P-VS6		
S1		DEK2	M	5/1/10	7.2	+	-	-		SHIV	8	8		DEK2-S1		
S2		R27	M	4/25/10	6.5	+	-	-		SHIV	8	8		R27-S2		
S3	Naïve- SHIV	R51	F	5/1/08	6.1	+	-	-		No VL	8	8			R51-S3	
S4		R59	M	5/2/08	7.6	+	-	-		SIV	5	5		R59-S4		R59-S4
S5		861	M	5/8/08	9.3	+	-	-		No VL	8	8			861-S5	861-S5
S6		824/KMV	F	8/1/09	4.8	-	+	-	-	No VL	8		8		824-S6	824-S6
N1		0FI	F	1/1/13	3.3	-				SIV	1		1			OF1-N1
N2		05D	F	1/1/13	3.4	-				SIV	2		2			O5D-N2
N3	Naïve	DFXM	M	3/25/13	3.5	-				SIV	6		6			DFXM-N3
N4		DF2B	M	5/20/13	3.3	+				SIV	6	6				
N5		DF3T	M	4/12/13	3.5	+				SIV	2	2				
N6		DFKL	M	1/1/12	4.1	-				No VL	8		8			

Supplementary Table 3. The Spearman's correlation between challenge times and immune tolerance genes/other genes

Challenge times VS.	BYSL	PDGFA	ADAR	ALPK1	CIITA	CYBB	IRF8	PNPT1	PSMD5	SPP1	CSF2RB
Spearman r											
r	-0.76	0.77	-0.70	-0.65	-0.66	-0.63	-0.71	-0.60	-0.60	0.63	-0.47
P value											
P (two-tailed)	0.0003	0.0006	0.0012	0.0033	0.0029	0.0050	0.0011	0.0086	0.0091	0.0086	0.0479
P value summary	***	***	**	**	**	**	**	**	**	**	*
Significant? (alpha = 0.05)	Yes	Yes	Yes	Yes	Yes	Yes	Yes	Yes	Yes	Yes	Yes
Challenge times VS.	CSK	GAB2	OAS3	OASL	PML	SEMA7A	IL22	LGALS3BP	NLRP1	RSAD2	THEMiS2
Spearman r											
r	-0.58	-0.51	-0.51	-0.55	-0.57	-0.57	-0.35	-0.28	0.30	-0.31	-0.37
P value											
P (two-tailed)	0.0118	0.0295	0.0303	0.0171	0.0129	0.0145	0.1579	0.2518	0.2345	0.2090	0.1319
P value summary	*	*	*	*	*	*	ns	ns	ns	ns	ns
Significant? (alpha = 0.05)	Yes	Yes	Yes	Yes	Yes	Yes	No	No	No	No	No

Supplementary Table 6. Antibodies used in this study.

Antibody	conjugated	clone	company
CD3	PE-Cy7	SP34-2	BD
CD4	BV605	L200	BD
CD8	APC-Cy7	RPA-T8	BD
CD28	FITC	CD28.2	Biolegend
CD95	PE-Cy5	DX2	Biolegend
IFNγ	PE or PerCP	4S.B3	Biolegend
CM9-dextramer	PE		ImmuDex
CD62P	PE	KPL-1	BD
CD41	FITC	HIP8	Biolegend
PF4	APC	Rabbit poly-clone, Cat#500-P05	Peprotech & Novus
CD14	Brilliant Violet 711™	M5E2	Biolegend
CD16	Brilliant Violet 421™	3G8	Biolegend
CD45	PerCP-Cy5.5	D058-1283	BD
CD8 depletion Ab		MT807R1	https://www.nhpreagents.org/ Nonhuman Primate Reagent Resource
